# Detour Behavior of Mice Trained with Transparent, Semitransparent and Opaque Barriers

**DOI:** 10.1371/journal.pone.0162018

**Published:** 2016-09-02

**Authors:** Grzegorz R. Juszczak, Michal Miller

**Affiliations:** Department of Animal Behavior, Institute of Genetics and Animal Breeding, Jastrzebiec, Poland; University of Queensland, AUSTRALIA

## Abstract

Detour tasks are commonly used to study problem solving skills and inhibitory control in canids and primates. However, there is no comparable detour test designed for rodents despite its significance for studying the development of executive skills. Furthermore, mice offer research opportunities that are not currently possible to achieve when primates are used. Therefore, the aim of the study was to translate the classic detour task to mice and to compare obtained data with key findings obtained previously in other mammals. The experiment was performed with V-shaped barriers and was based on the water escape paradigm. The study showed that an apparently simple task requiring mice to move around a small barrier constituted in fact a challenge that was strongly affected by the visibility of the target. The most difficult task involved a completely transparent barrier, which forced the mice to resolve a conflict between vision and tactile perception. The performance depended both on the inhibitory skills and on previous experiences. Additionally, all mice displayed a preference for one side of the barrier and most of them relied on the egocentric strategy. Obtained results show for the first time that the behavior of mice subjected to the detour task is comparable to the behavior of other mammals tested previously with free-standing barriers. This detailed characterization of the detour behavior of mice constitutes the first step toward the substitution of rodents for primates in laboratory experiments employing the detour task.

## Introduction

Detour tasks are commonly used to study problem solving skills and inhibitory control in mammals, especially in canids, monkeys and human infants [1–3]. Currently used tasks derive from pioneering observations made by Köhler in dogs and chickens [4]. He noticed that an apparently simple task requiring a dog to move around a small wire fence was in fact very difficult when a highly desired object was placed directly behind the barrier. In such a situation “the very nearness” of the target blocked the ability to find the solution and, as a result, the dog kept pushing against the fence instead of moving around it [4]. The most important feature of this task was the visibility of the target that was placed directly behind the barrier, the fact that the entire field of possible detours was in plain sight and the necessity to move away from the target to reach the goal [4]. Therefore, the performance on the task depended on the ability to inhibit prepotent and counterproductive responses driven by the visual stimulus. Since the times of Wolfgang Köhler similar behavioral deficits have been found in different species faced with this apparently simple problem [3,5–7]. Currently used detour tasks that are based on the classic concept often differ in some details from the original design applied by Köhler. They require the movement of the entire body (locomotor test) or only the movement of the arm (reaching test) [8] and employ barriers taking different shapes (I, L, U, V shaped) [8–11] or clear acrylic boxes open from one or more sides [7,12]. However, all these versions of the task meet the aforementioned basic requirements proposed by Köhler.

Detour tests gained popularity because they have many different applications. First, they have been used to study development of executive skills in normal [7,8] born preterm [13] and autistic children [14] and in infant monkeys [7]. Second, pharmacologically-induced impairments in monkeys’ performance on the detour task are used to model cognitive deficits observed in various neurodegenerative diseases [15–18], schizophrenia and drug abuse [19–24]. Furthermore, there is also an increasing interest in the neuronal mechanism responsible for solving the detour problem in navigation [25]. However, despite the significance of the detour task for basic and applied research, there is no comparable detour task designed for rodents. It also means that there is no study enabling the comparison of rodent detour behavior with other mammals that were previously extensively tested with the classic detour task based on the concept developed by Köhler [4]. The only rodent study that employed free-standing barriers was performed in rats and was based on climbing behavior motivated by food [26]. However, the tasks designed by Jovalekic et al. [26] were not meant to study the executive functions such as inhibitory skills but instead were designed to study navigation in two- and three-dimensional environments with a vertical dimension. Therefore, these tasks did not meet the requirements of classic detour task such as presence of well visible target placed directly behind the barrier. Therefore, the aim of the present study was to translate the classic detour task to mice. The experimental design combined all the most important methodological developments such as application of outward and inward detour trials that were used previously in dogs and dingoes [1,11] and usage of barriers differing in transparency [5,6,8]. In order to validate the paradigm, we checked whether the mouse detour task would allow us to recapitulate the key behavioral findings obtained in other mammals. More specifically, we wanted to check both the effect of target visibility and previous experiences on the detour behavior. Such experiments were performed previously in human infants [2,7,8], monkeys [5,17] and some birds [6]. Second, we were interested in directional preference and navigation mode of mice subjected to the detour task. Such experiments were performed previously only on dingoes [1]. Obtained data show that the detour behavior of mice is comparable with the behavior of other previously tested species.

## Materials and Methods

### Animals

The subjects were thirty six F1 hybrid (C57BL10 x CBA/H) male mice obtained from the breeding colony located at the Institute of Genetics and Animal Breeding (Jastrzebiec). Animals were marked with ear notches (1 week before the experiment) and moved to the testing room two days before the beginning of the experiment. The mice were 15 weeks old and weighed 30.7 ± 0.4g (mean ± SEM) at the beginning of the experiment. The mice were group housed (4–7 per cage) under standard laboratory conditions (12:12 h light/dark cycle, relative humidity of 45% and ambient room temperature of 22°C) with standard murine chow and water available *ad libitum*. Standard laboratory mouse cages (207 mm × 265 mm and 140 mm high) were made of clear polycarbonate and were covered with stainless steel wire-grid lids that held feed and water bottle. Softwood granules were used as a bedding material. Each group was tested with different kind of barrier (transparent, semitransparent or opaque) and initially counted 12 mice. Two mice (1 from transparent and 1 from semitransparent group) had to be removed from the final analysis because of an error in changing the barrier that occurred at the beginning of the training. Therefore, the final number of animals in the transparent and semitransparent group was eleven.

The mice were under constant veterinarian care and all procedures were performed in accordance with the Guiding Principles for the Care and Use of Research Animals. The study has been approved by the Warsaw Third Local Ethical Committee for Animal Research, which is responsible for the supervision of animal research performed in the Institute of Genetics and Animal Breeding (Permit Number: 34/2014, according to Polish Ministry of Agriculture and Rural Development decree from 10.03.2006 on conditions of maintaining laboratory animals). All efforts were made to minimize the animals’ suffering.

### Water escape detour test

#### Apparatus

**Fig 1:**
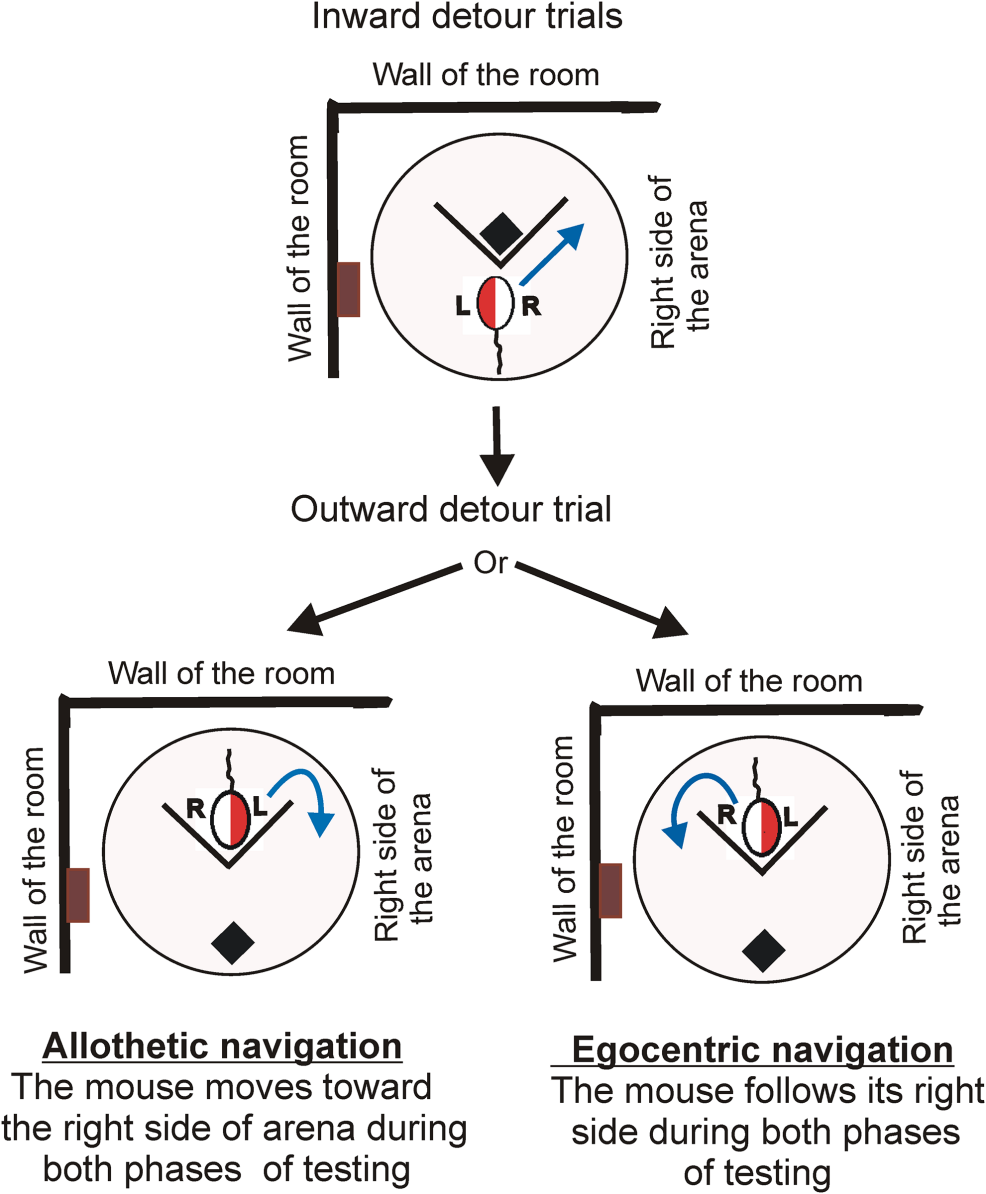
Determination of the navigation strategy. The blue arrows show the preferred direction of movement. R and L means right and left side of the mouse determined based on the body-centered coordinates. The right side of the arena is determined based on the room coordinates. The brown rectangle depicts a vertical wooden post that constituted a landmark together with two walls adjoining the tank.

**Fig 2:**
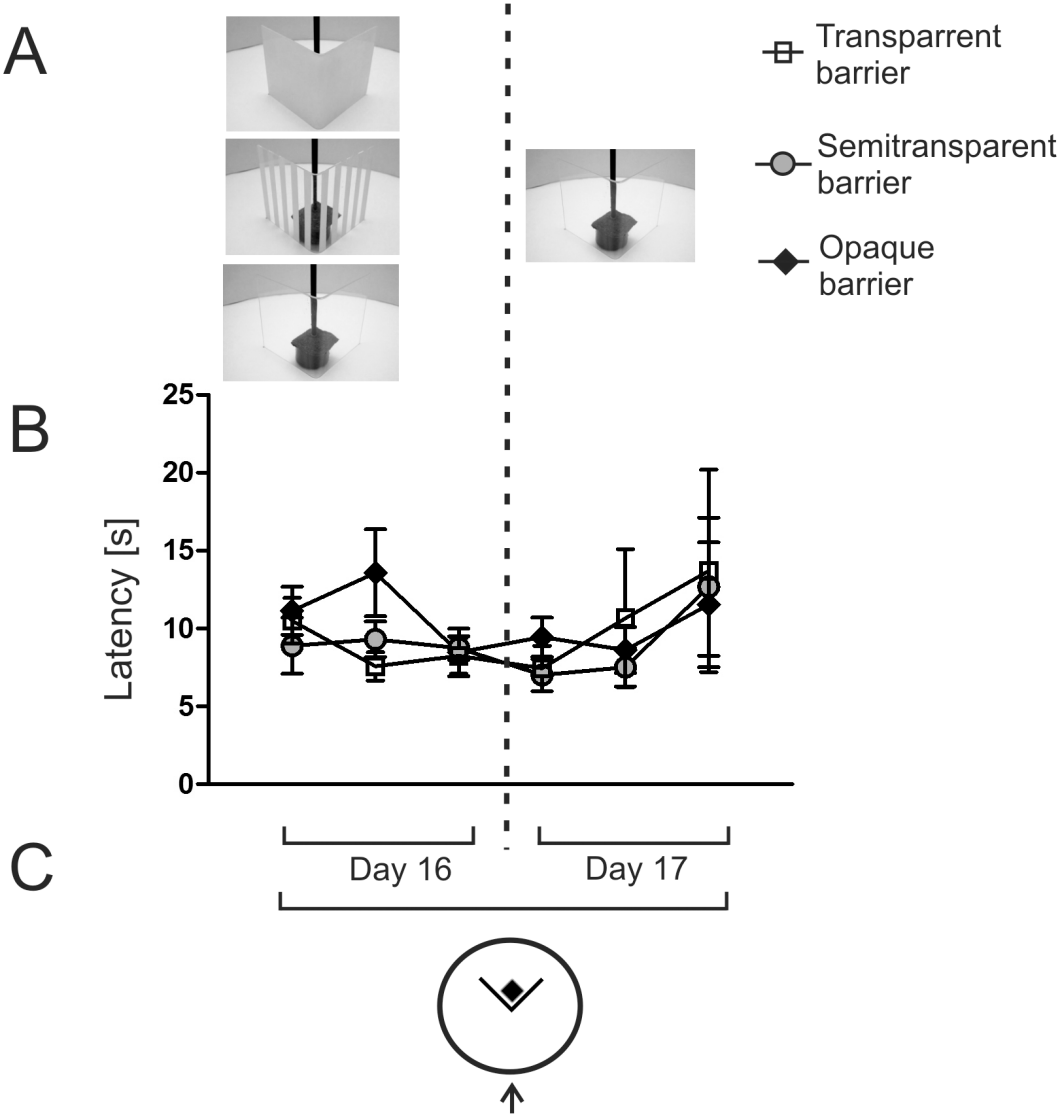
Effect of the previous experience with different barriers on the ability to detour the transparent barrier. Animals that were used in the first part of the experiment (Fig 3) were retrained to detour the same kind of barrier as previously (left side) and next were tested with the transparent barrier (right side). A—picture of opaque, semitransparent and transparent barrier together with the escape platform. B—latency to reach the platform placed behind the barrier. C—diagram presenting the experimental design. The arrow shows the starting point. Values are presented as mean ± SEM.

To motivate animals to perform the detour task, we used the water escape paradigm, which is one of the most successful approaches used in rodent behavioral models [27,28]. The apparatus consisted of a white circular tank (28 cm high and 96 cm in diameter) filled with water (24.5 ± 1.5°C) that was 5.5 cm deep. This depth of water allowed the mice to stand on their back legs and thus enabled a rest and decreased the stress associated with the test. The apparatus was painted white to enable automated video tracking that is based on the contrast between the tracked object and the background. The tank was placed in a corner of the experimental room so that two sides of the tank adjoined the walls of the room while two other sides were surrounded with open space. Additionally, there was a vertical wooden post (4 cm wide) that was attached to the wall (Fig 1). The post was used as a support for a video camera positioned above the pool but it also constituted a well visible landmark. Therefore, it was possible to distinguish between left, right, front and back side of the tank depending on the position of the barrier within the tank and position of the walls of the experimental room (Fig 1). The platform (Fig 2a, S1 File) consisted of a round plaster cast (5.5 cm high and 7.5 cm in diameter) that was painted black and covered with a square piece of dark gray polyurethane foam having a shape of a clipped pyramid (8.5 cm wide at the base, 5 cm wide at the top and 2 cm high). The part made of polyurethane foam protruded above the water and provided surface suitable for climbing. A black metal rod (1.2 cm in diameter and 38 cm long) was placed in the central part of the platform to ensure that the location of the platform was easy to notice for swimming mice. To prevent animals from reaching their goal, we used a transparent, semitransparent and opaque barrier depending on the experimental group. All barriers were made out of one piece of clear acrylic glass, which was bent at an angle of 90 degrees (Fig 2a, S1 File). Additionally, we painted vertical white stripes on one of the barriers to make it semitransparent. The stripes were 1.8 cm wide and were spaced 1.8 cm apart. The opaque barrier was painted white on the entire surface (Fig 2a). Each arm of the barrier was 20 cm high and 18 cm wide.

#### Procedure

**Fig 3:**
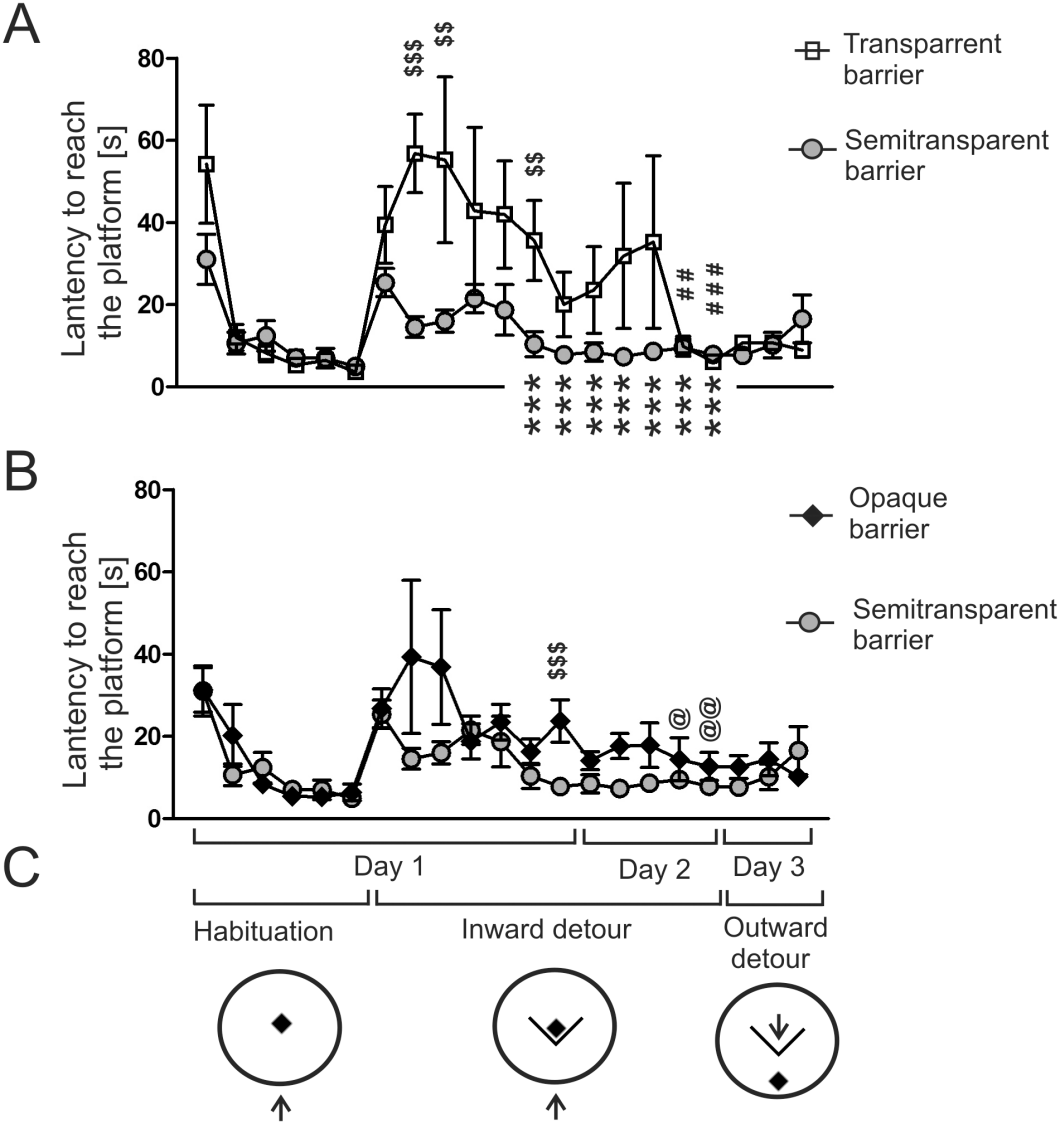
Performance of the mice subjected to the inward and outward detour test. A—transparent group compared with the semitransparent group. B—opaque group compared with the semitransparent group. C—diagram presenting the experimental design. The arrow shows the starting point. $ $ = *P* < 0.01, $ $ $ = *P* < 0.001—denotes a significant differences between groups for a given trial; ## = *P* < 0.01, ### = *P* < 0.001—denotes significant improvement in the transparent group compared with the first detour trial; *** = *P* < 0.001 –denotes a significant improvement in the semitransparent group compared with the first detour trial; @ = *P* < 0.05, @@ = *P* < 0.01 –denotes a significant improvement in the opaque group compared with the first detour trial. Values are presented as mean ± SEM.

The experiment was divided into two parts. The first part was performed during 3 consecutive days and started with habituation of the mice to the pool with a platform located in the centre of the tank without any barrier (S1 File). During the habituation period, the mice were placed in the pool and were allowed to swim until they climbed the platform. This procedure was repeated 6 times for each animal. The habituation period was followed by 7 inward detour trials performed on the same day, 5 inward detour trials performed on the second day and 3 outward detour trials performed on the third day (Fig 3c). The first detour trial was performed 30 min after the end of the last habituation session. The inward and outward detour trials were based on the procedure used previously to test behavior of dingoes and domestic dogs [1,11]. During the habituation period and inward detour trials, the mice were placed in the pool always at the same location near the wall with their heads facing the platform, which was located 56 cm from the starting point and 46 cm from the left and right side of the tank. During inward detour trials, the mice were separated from the target with a barrier that touched the platform (S1 File). During outward detour trials, the position of the platform and the starting point were exchanged, while the position of the barrier was not altered (Fig 3c, S1 File). Depending on the group, the mice were tested with transparent, semitransparent or opaque barrier. Application of the inward and outward detour trials allowed us to distinguish between a strategy based on the position of environmental landmarks and a strategy based on the body-centered coordinates (Fig 1). The displacement of the target during outward trials allowed us also to check whether mice display a learned sequence of movements (swimming around the barrier) or orient towards the new position of the target.

The second part of the experiment started after about 2 weeks of rest (the median number of days between the first and second part was 12) and only employed the inward detour paradigm. On the first day after the rest period, the mice were retrained to detour the transparent, semitransparent or opaque barrier (Fig 2a and 2c). The retraining phase consisted of 3 trials and the composition of groups was the same as in the first part of the experiment. On the second day, all mice were tested three times with the transparent barrier to check whether the mice that have already learned to detour the opaque or semitransparent barriers will have difficulty in detouring the completely transparent barrier.

During all detour trials, the mice were allowed to swim until they found the platform or until 4 min passed. After each trial, the mouse was placed in a transportation cage and returned to the home cage for the intertrial period. Both the transportation cage and the home cage were lined with paper towels. The mice were trained and tested in groups of 4–7 animals belonging to the same litter. Mice from each litter were assigned to all experimental groups in a pseudorandom way. The rest periods between trials were equal to one round of trials performed on all other mice from the same cage and ranged between 11.8 ± 1.3 min (mean ± SEM) at the beginning of the training and 10.5 ± 1.0 min (mean ± SEM) at the end of the training. These rest periods correspond well with intertrial intervals of 10 min advocated for testing mice in the Morris water maze [27]. The behavioral testing was performed from 10 am to 4 pm during the light phase of the animals' light:dark cycle.

### Video tracking

The test was recorded with a video camera positioned above the pool and analyzed with the EthoVision system (EthoVision 3.1, Noldus Information Technology, Wageningen, The Netherlands) [29]. The EthoVision software was used to define a barrier zone and to record animals’ paths (tracks). The zone was used to measure perseveration during inward detour trials. Graphical representations of animals’ paths were used to measure path deviation from the central axis of the barrier. All measurements were rescaled to represent the real distances within the experimental arena. If necessary, the number of displayed points that belonged to the track was restricted to obtain a clear view of animal’s path during initial approach to the target (the first approach after the mouse was placed in the pool).

The analysis was preceded by preliminary comparison of the EthoVision tracks and videos. This comparison enabled us to choose a proper width of the zone and a proper distance between the barrier and the reference line used for the analysis of tracks. The position of an animal is determined by the EthoVision software based on the position of the mathematical centre of the tracked object [29,30]. Therefore, the tracks are located at some distance from the barrier even when the body of the mouse touched the barrier. The comparison of the EthoVision tracks and videos revealed that the maximal distance between the animal’s path and the barrier was 3.7 cm at the time when the mouse touched the barrier with its head after approaching it at a straight angle. Therefore, the applied distance of 5 cm between the reference line and the barrier was slightly larger than the maximal distance between the path and the barrier at the time when an animal could touch the obstacle. The width of the barrier zone (5 cm) was also based on this assessment.

### Perseveration

**Fig 4:**
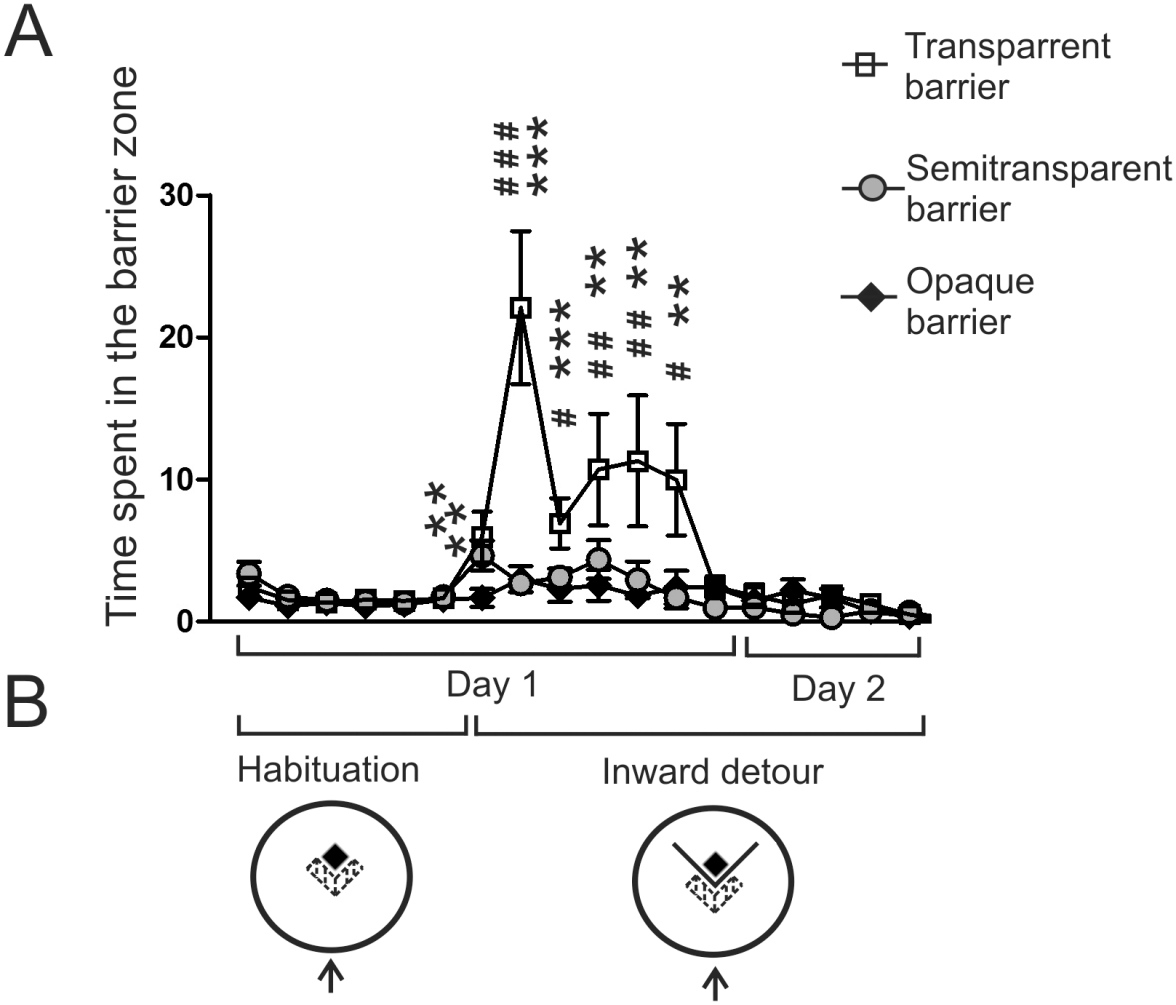
Perseveration of the mice tested on the detour task. A—time spent in the barrier zone. B—diagram presenting the experimental design. The arrow shows the starting point. A hatched area depicts the barrier zone. # = *P* < 0.05, ## = *P* < 0.01, ### = *P* < 0.001—denotes a significant increase compared with the last habituation session; ** = *P* < 0.01, *** = *P* < 0.001—denotes significant differences compared with the opaque group for a given trial. Values are presented as mean ± SEM.

Perseveration is defined as a tendency to respond persistently to a particular stimulus, even after the response has become inappropriate [31]. In the detour test, perseverative behaviors included scratching the barrier and persistent returning to the place located in front of the barrier. Both these behaviors were assessed jointly by measurement of the total time spent in the barrier zone which was located in front of the barrier and was V shaped (Fig 4b). Each arm of the barrier zone was 8.5 cm long (internal length at the border with the barrier) and 5 cm wide. The barrier zone covered the area directly in front of the platform placed behind the barrier.

### Direction of movement during the initial approach to the target

**Fig 5:**
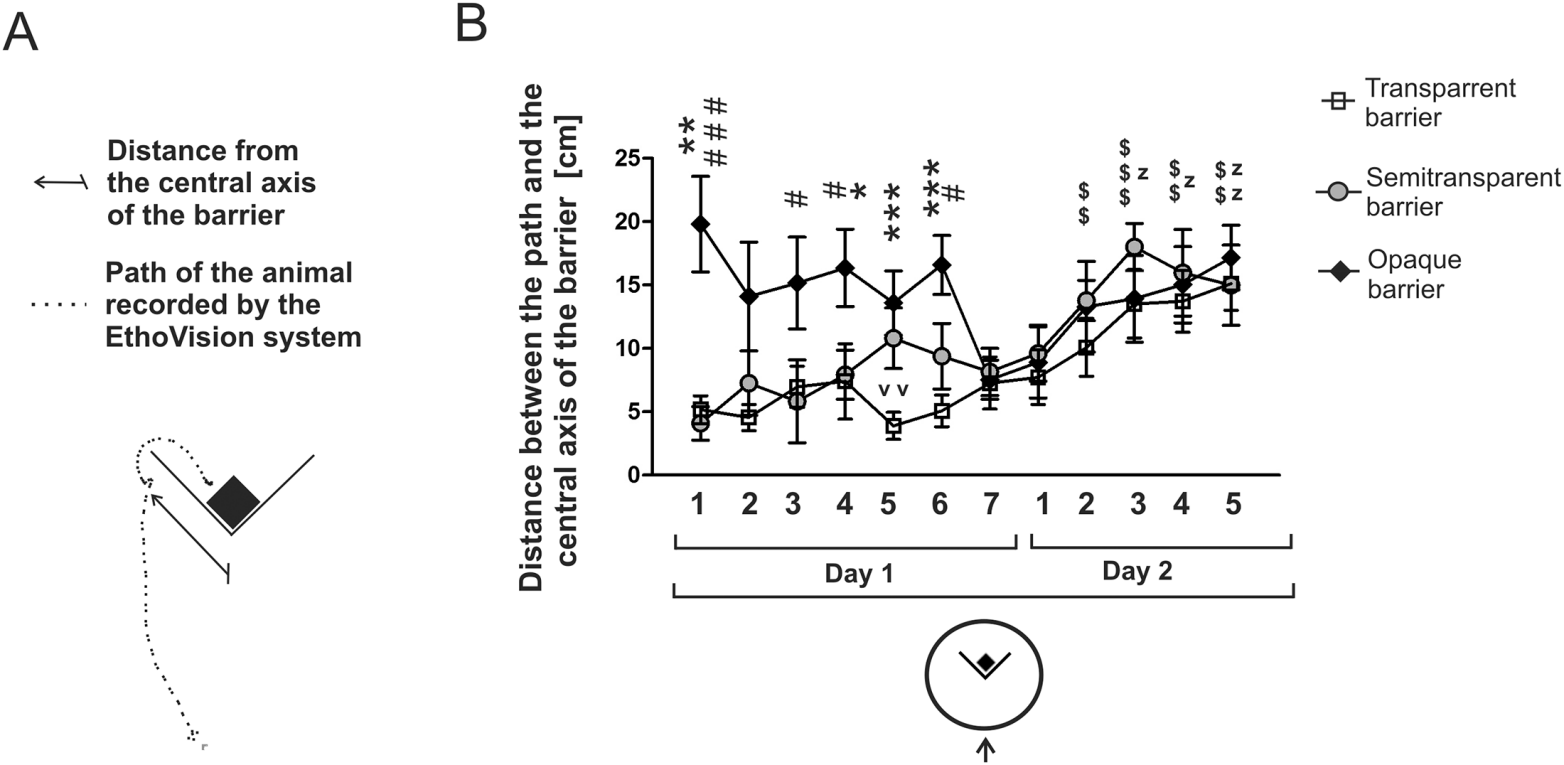
Distance between the animal’s path and the central axis of the barrier during the initial approach to the target. A—diagram presenting measurement of the distance. B—data obtained during the first two days of the experiment (inward detour trials). * = *P* < 0.05, ** = *P* < 0.01, *** = *P* < 0.001—denotes significant differences between the opaque and transparent group; # = *P* < 0.05, ### = *P* < 0.001—denotes significant differences between the opaque and semitransparent group; v v = *P* < 0.01—denotes a significant differences between the transparent and semitransparent group; z = *P* < 0.05, zz = *P* < 0.01—denotes significant change in the transparent group compared with the first trial (day 1); $ $ = *P* < 0.01, $ $ $ = *P* < 0.001—denotes significant differences in the semitransparent group compared with the first trial (day 1). Values are presented as mean ± SEM.

Assesment of the direction of movement during the initial approach to the target allowed us to estimate both the effect of target visibility on behavior and the ability to optimize the direction of movement based on the experiences from previous trials. Therefore, we measured the distance between the central axis of the barrier and the animal’s path at a point where the path crossed for the first time the reference line that was parallel to the barrier and was located 5 cm from its left or right arm (Fig 5a). The distance between the reference line and the barrier (5 cm) was determined based on the aforementioned comparison of the EthoVision tracks and videos and was similar (slightly larger) to the maximal distance between the path and the barrier at the time when the animal could touch the obstacle.

### Statistics

Variance homogeneity and sphericity were assessed with Hartley's and Mauchly's test respectively. Data used for the repeated measures two-way ANOVA did not meet the requirement of sphericity and therefore we applied the multivariate approach to repeated measures ANOVA (MANOVA) with Wilks' Lambda test. To assess the effect of training, we used two-tailed Dunnett's test performed separately for each group. Detailed between-group comparisons have been done with one-way ANOVA followed by Fisher least significance difference (LSD) test. Data that did not meet the requirement of variance homogeneity or sphericity (one-way ANOVA and post hoc test for repeated measurements) were subjected to logarithmic transformation following the data normalization guidelines [32]. Datasets that contained some zero values were transformed using following formula: *x* = log(*y* +*c*), where *c* is a constant = 1 and y is the transformed value [32]. Data analysis was performed with Statistica software, release 7.1. Values are presented as mean ± SEM.

## Results

### Habituation and inward detour

During the habituation period, animals were trained to use the platform without the barrier (Fig 3). During the first habituation session, mice tended to ignore the platform located in the centre of the tank and instead explored the wall of the tank before finally climbing the platform. This pattern of behavior changed during subsequent sessions because mice learned quickly to swim directly to the platform. Multivariate two-way ANOVA revealed a significant effect of the habituation session (*F*5,27 = 7.91, *P* = 0.0001), while the effect of group and the interaction were not significant (*F*2,31 = 0.49, *P* = 0.6 and *F*10,54 = 1.0, *P* = 0.45, respectively). The lack of significant differences between groups has been confirmed by one-way ANOVA performed separately for each habituation session (*F*2,31 = 0.85, *P* = 0.44 (session 1); *F*2,31 = 1.19, *P* = 0.32 (session 2); *F*2,31 = 0.77, *P* = 0.47 (session 3); *F*2,31 = 0.40, *P* = 0.67 (session 4); *F*2,31 = 0.23, *P* = 0.80 (session 5); *F*2,31 = 0.99, *P* = 0.38 (session 6)). All groups significantly improved their performance (Dunnett's test) during the 3^rd^ (at least *P* < 0.01), 4^th^ (at least *P* < 0.0001), 5^th^ (at least *P* < 0.0001), and 6^th^ (*P* < 0.00001) habituation session compared with the first session (significance levels not marked on Fig 3). The video presenting the behavior during habituation phase and examples of tracks are available in S1 File and S1 Fig.

**Fig 6:**
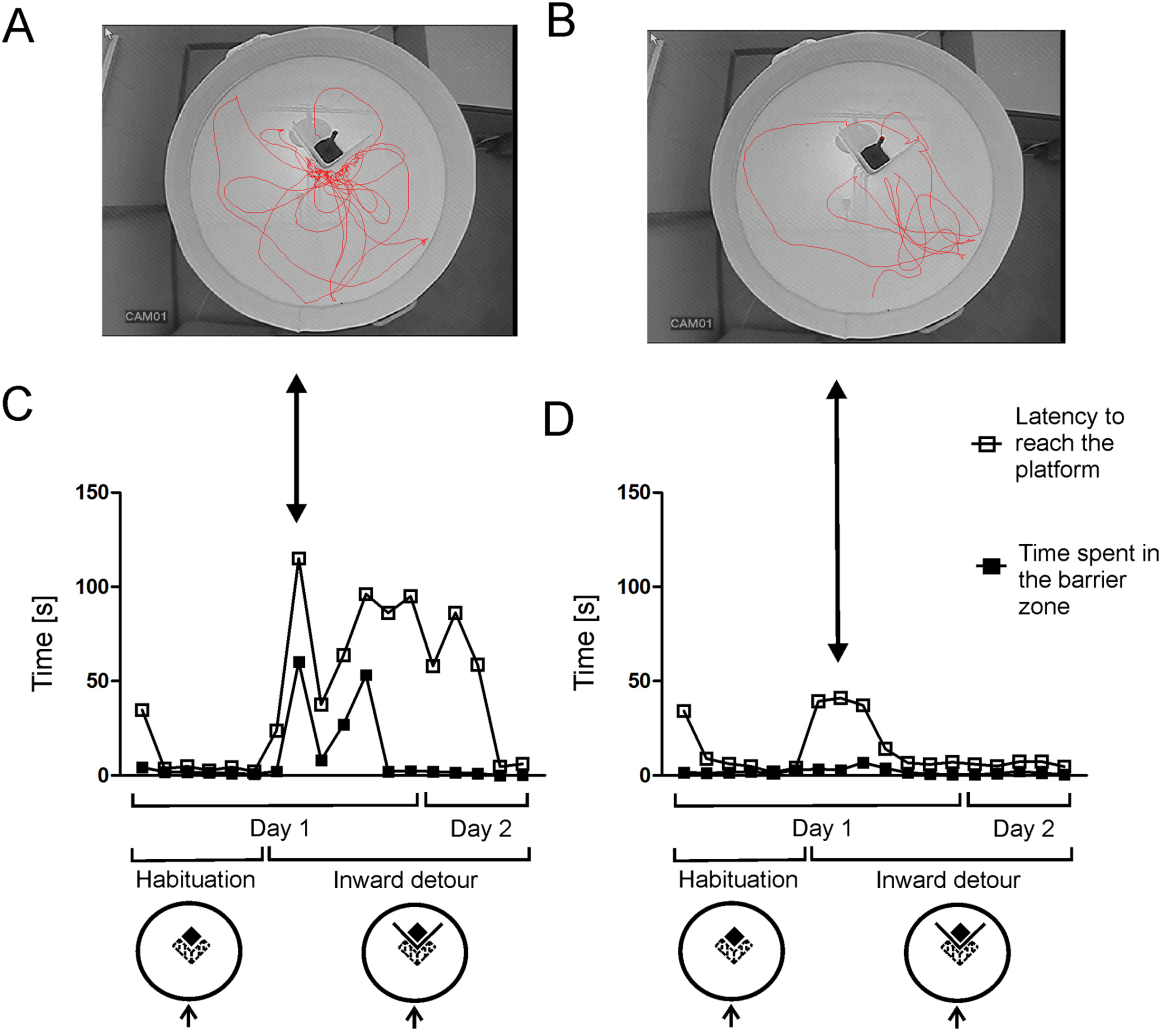
Individual performance of the mice from the transparent group. A and B show tracks of mice displaying a high and low level of perseveration, while C and D shows overall performance of these mice. Large arrows mark data collected from the same mice and identify detour trials depicted in panel A and B, small arrows show the starting point, a hatched area depicts the barrier zone.

The latencies increased rapidly in all groups when the barrier was placed in front of the platform (Fig 3). At the beginning, the mice repeated the pattern of swimming toward the barrier and away until they found the open side of the platform (Fig 6a and 6b, S1 File and S1 Fig). During subsequent trials, the mice gradually learned the task and improved their performance as indicated by shortened latencies (Fig 3). Only 3 mice (transparent and opaque group) failed to detour the barrier during 1 or maximally 2 trials. In one case (one trial), the mouse managed to jump onto the barrier but never repeated this feat during subsequent trials. It is worth mentioning that no mouse managed to jump onto the barrier during the preliminary experiment that was done to test the experimental setup. Multivariate two-way ANOVA (calculated for the inward detour trials performed on the first and second day) revealed a significant effect of trial (*F*11,21 = 6.56, *P* = 0.0001) and experimental group (*F*2,31 = 5.53, 0.009) while the interaction was not significant (*F*22,42 = 1.34, *P* = 0.20). A post-hoc analysis (Dunnett's test) revealed that all groups improved significantly their performance although the mice needed different number of trials depending on the applied barrier (Fig 3). The mice trained with the semitransparent barrier improved significantly during the sixth detour trial performed on the first day (*P* = 0.0001) and maintained good performance during all subsequent inward trials (*P* = 0.00004 (7^th^ trial, day 1), *P* = 0.00002 (1^st^ trial, day 2), *P* = 0.00002 (2^nd^ trial, day 2), *P* = 0.0001 (3^rd^ trial, day 2), *P* = 0.0004 (4^th^ trial, day 2), *P* = 0.00003 (5^th^ trial, day 2)). Two other groups required two days of training to improve significantly their performance (Fig 3). The mice trained with the transparent barrier improved significantly during the 4^th^ (*P* = 0.002) and 5^th^ (*P* = 0.00006) trial performed on the second day (Fig 3). Similarly, the mice trained with the opaque barrier improved significantly their performance during the 4^th^ (*P* = 0.01) and 5^th^ (*P* = 0.006) trial performed on the second day (Fig 3).

During the first detour trial (Fig 3) there were no differences between groups (*F*2,31 = 1.03, *P* = 0.37, one-way ANOVA). Significant differences appeared during the 2^nd^, 3^rd^, 6^th^, and 7^th^ trial performed on the first day (*F*2,31 = 9.15, *P* = 0.0007, *F*2,31 = 4.58, *P* = 0.02, *F*2,31 = 4.61, *P* = 0.02, *F*2,31 = 6.90, *P* = 0.003 respectively). During the second day of training, the differences were not significant, although during the 2^nd^ trial the effect of the experimental group approached the level of significance (*F*2,31 = 2.81, *P* = 0.076). A post-hoc analysis (Fisher's test) revealed that the mice tested with the transparent barrier displayed significantly longer latencies compared with the semitransparent group during the 2^nd^, 3^rd^ and 6^th^ trial performed on the first day (*P* = 0.0002, 0.005 and 0.005 respectively; Fig 3). The mice tested with the opaque barrier displayed significantly longer latencies compared with the semitransparent group during the 7^th^ trial (*P* = 0.0008, Fig 3). The video presenting the behavior of the mice subjected to the inward detour task and examples of tracks are available in S1 File and S1 Fig.

In addition to the between-group differences, there were also large differences in latencies between individual animals trained with the transparent barrier (Fig 6). These differences resulted in part from variability in perseveration, which was especially large at the beginning of the training (see the next section). Additionally, large variability, especially during the second day of training, resulted from long latencies displayed by two mice that repeatedly caught the edge of the transparent barrier and stayed in this position for a prolonged period of time (30 s or more).

### Perseveration

In order to assess perseveration in the mice, we have measured the time spent in the barrier zone located in front of the barrier (Fig 4). Multivariate two-way ANOVA that included data from the last session of the habituation period (for comparison) and all inward detour trials performed on the first and second day showed that there was a significant effect of trial (*F*12,20 = 11.28, *P* = 0.000002), experimental group (*F*2,31 = 19.10, *P* = 0.000004) and also significant interaction (*F*24,40 = 5.01, *P* = 0.000004). A post-hoc analysis (Dunnett's test) showed that there was significant increase in the time spent in the barrier zone only in the group of mice tested with the transparent barrier during the 2^nd^ (*P* < 0.00001), 3^rd^ (*P* = 0.03), 4^th^ (*P* = 0.007), 5^th^ (*P* = 0.005) and 6^th^ (*P* = 0.03) trial performed on the first day of the experiment (Fig 4). One-way ANOVA revealed a significant effect of group during the 1^st^ (*F*2,31 = 6.05, *P* = 0.006), 2^nd^ (*F*2,31 = 16.45, *P* < 0.00001), 3^rd^ (*F*2,31 = 6.75, *P* = 0.004), 4^th^(*F*2,31 = 4.68, *P* = 0.02), 5^th^ (*F*2,31 = 7.26, *P* = 0.003), and 6^th^ (*F*2,31 = 5.69, *P* = 0.008) trial. A post-hoc analysis (Fisher's test) revealed that the mice tested with the transparent barrier spent significantly more time in the barrier zone compared with the opaque group during the 1^st^ (*P* = 0.004), 2^nd^ (*P* = 0.00001), 3^rd^ (*P* = 0.0009), 4^th^ (*P* = 0.004), 5^th^ (*P* = 0.001) and 6^th^ (*P* = 0.007) trial (Fig 4). The mice tested with the transparent barrier also spent significantly more time in the barrier zone compared with the semitransparent group during the 2^nd^ (*P* = 0.00004), 3^rd^ (*P* = 0.04), 5^th^ (*P* = 0.007) and 6^th^ (*P* = 0.006) trial (significance levels not marked on Fig 3). The differences between the semitransparent and opaque group were small and turned out to be significant only during the 1^st^ trial (*P* = 0.008). In addition to the between-group differences in perseveration, there were also large differences in perseveration between individual animals in the transparent group (Fig 6). The video presenting the behavior of mice subjected to the inward detour task and examples of tracks are available in S1 File and S1 Fig.

### Outward detour

The mice that had experience with the inward detour task performed well during the outward detour test (Fig 3). Average latencies were below 17 s in all groups and were similar to latencies during two last trials of the inward detour task (Fig 3). Multivariate two-way ANOVA that included data from the last inward detour trial (for comparison) and 3 outward detour trials showed an insignificant effect of trial (*F*3,29 = 1.40, *P* = 0.26) and experimental group (*F*2,31 = 0.67, *P* = 0.52) although there was a significant interaction (*F*6,58 = 2.36, *P* = 0.042). A post-hoc analysis (Dunnett's test) showed that there was significantly longer latency in the group of mice tested with the transparent barrier during the 1^st^ and 2^nd^ outward trial compared with the last inward trial (*P* = 0.01; significance levels not marked on Fig 3). Although these differences were significant, the effect size was very small because mean latencies during the 1^st^ and 2^nd^ outward trial were only 4.6 s longer than during the last outward trial. Differences between the last inward trial and outward trials were not significant in other groups. There were also no differences between groups during consecutive outward trials.

During the outward trials, the platform was placed 56 cm behind the barrier, in contrast to the inward trials performed with the platform touching the barrier. Usually, the mice swam directly toward the platform placed in a new location after finding the edge of the barrier instead of turning and swimming along the wall toward the central part of the barrier where previously the platform was located. During the first outward trial, such goal-directed behavior was displayed by 8 mice from the semitransparent group, 10 mice from the transparent group and 11 mice from the opaque group. Only in 5 cases the mice subjected to the outward detour task followed the wall of the barrier and returned to the starting point. This happened almost exclusively in the group of mice tested with the semitransparent barrier (4 cases) and mainly during the last trial of the outward detour test (4 cases). An exception was one mouse that swam around the barrier and returned to the starting point during the second trial. The video presenting the behavior of a mouse subjected to the outward detour task and examples of tracks are available in S1 File and S1 Fig.

### Effect of the previous experience with the opaque and semitransparent barrier on the ability to detour the transparent barrier

After 2 weeks of the rest period, the mice were retrained to detour barriers using the inward paradigm (Fig 2). The mice were trained with the same kind of barrier as previously (transparent, semitransparent or opaque). Despite the rest period the mice solved the task easily because mean latencies were below 14 s in all groups. On the next day, all mice were tested with the transparent barrier (Fig 2). Multivariate two-way ANOVA that included data from the last trial of the retraining phase (for comparison) and 3 trials performed only with the transparent barrier showed that there was no effect of trial (*F*3,29 = 1.61, *P* = 0.21) and experimental group (*F*2,31 = 0.06, *P* = 0.94) and a lack of significant interaction (*F*6,58 = 0.99, *P* = 0.44).

### Path direction

**Fig 7:**
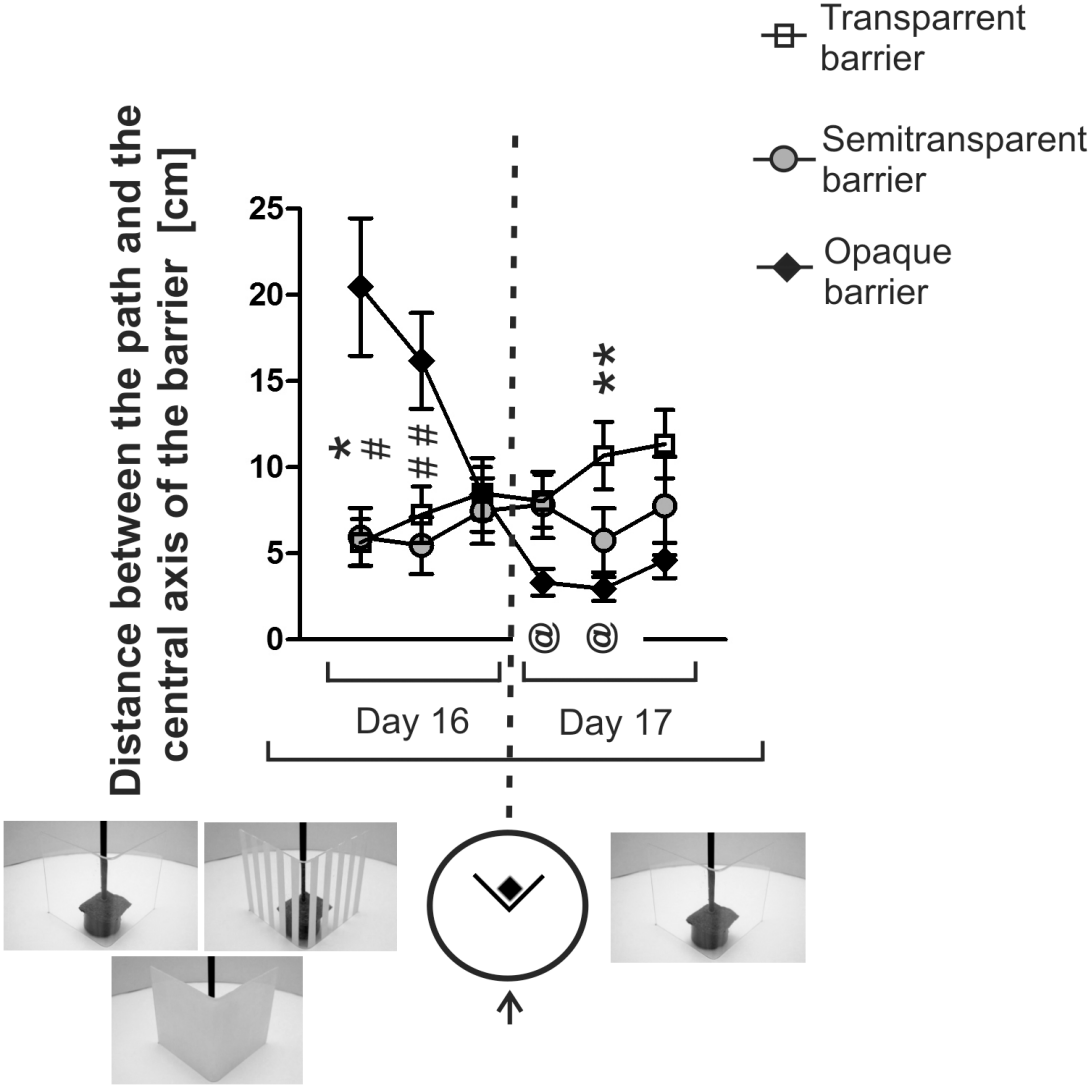
Distance between the animal’s path and the central axis of the barrier during initial approach to the target in animals retrained after the rest period (left side) and next tested exclusively with the transparent barrier (right side). * = *P* < 0.05, ** = *P* <0.01—denotes significant differences between the opaque and transparent group for a given trial; # = *P* < 0.05, ## = *P* < 0.01—denotes significant differences between the opaque and semitransparent group; @ = *P* < 0.05—denotes a significant change in the opaque group compared with the last trial of the retraining phase. Values are presented as mean ± SEM. For more explanations see Fig 5.

The direction of movement may indicate whether mice plan in advance the action to optimize the length of the path. In order to assess the path direction during the initial approach to the target, we have measured the distance between animal’s path and the central axis of the barrier (Fig 5a). A separate analysis has been performed on data obtained during the initial inward detour training (Fig 5b) and inward detour trials performed after the rest period (Fig 7). Multivariate two-way ANOVA calculated for the detour trials performed on the first and second day revealed that there was a significant effect of trial (*F*11,21 = 3.05, *P* = 0.01) and experimental group (*F*2,31 = 12.61, *P* = 0.0001) while the interaction was not significant (*F*22,42 = 0.79, *P* = 0.71). A post-hoc analysis (Dunnett's test) revealed that the direction of movement changed significantly during consecutive trials in the transparent and semitransparent group, while in the opaque group the differences were not significant (Fig 5b). The mice trained with the semitransparent barrier changed significantly the direction of movement during the 2^nd^ (*P* = 0.002), 3^rd^ (*P* = 0.0001), 4^th^ (*P* = 0.006), and 5^th^ trial (*P* = 0.009) performed on the second day (Fig 5b). The mice trained with the transparent barrier changed significantly the direction of movement during the 3^rd^ (*P* = 0.02), 4^th^ (*P* = 0.02) and 5^th^ trial (*P* = 0.002) performed on the second day (Fig 5b).

The direction of movement of mice tested with the opaque barrier was different from the two other groups. The mice tested with the opaque barrier swam toward the left or right side of the tank after being placed in the pool, while the mice from the transparent and semitransparent group swam toward the platform visible behind the barrier. These differences, however, disappeared after a prolonged training (Fig 5b). One-way ANOVA revealed a significant effect of group during the 1^st^ (*F*2,31 = 11.98, *P* = 0.0001), 3^rd^ (*F*2,31 = 3.34, *P* = 0.04), 4^th^(*F*2,31 = 3.50, *P* = 0.04), 5^th^ (*F*2,31 = 8.69, *P* = 0.001), and 6^th^ (*F*2,31 = 7.48, *P* = 0.002) trial performed on the first day. A post-hoc analysis (Fisher's test) revealed that the direction of movement was significantly less divergent in the transparent group compared with the opaque group during the 1^st^ (*P =* 0.001), 4^th^ (*P* = 0.03), 5^th^ (*P* = 0.0004) and 6^th^ (*P* = 0.0006) trial (Fig 5b). The direction of movement was also significantly less divergent in the semitransparent group compared with the opaque group during the 1^st^ (*P* = 0.00006), 3^rd^ (*P* = 0.01), 4^th^ (*P* = 0.03), and 6^th^ (*P* = 0.02) trial (Fig 5b). The direction of movement of mice tested with the transparent and semitransparent barrier was very similar during almost all trials and significant differences occurred only during the 5^th^ trial performed on the first day (*P* = 0.004).

A similar pattern has been observed in the mice retrained after a rest period (day 16) although differences between the opaque and two remaining groups disappeared much faster (Fig 7). Differences between groups appeared again when all mice were tested exclusively with the transparent barrier (day 17) because animals having previous experience with the opaque barrier started to move toward the platform, while behavior of the mice having previous experience with the semitransparent barrier was intermediate compared with two other groups (Fig 7). Multivariate two-way ANOVA revealed that there was a significant effect of trial (*F*5,27 = 4.47, *P* = 0.004) and significant interaction (*F*10,54 = 6.01, *P* = 0.000005) while the effect of the group was not significant (*F*2,31 = 1.70, *P* = 0.20). A post-hoc analysis (Dunnett's test) revealed that only the mice having previous experience with the opaque barrier significantly change the direction of movement during the 1^st^ (*P* = 0.03) and 2^nd^ (*P* = 0.01) trial performed exclusively with the transparent barrier compared with the 3^rd^ trial performed during the retraining phase (Fig 7). One-way ANOVA revealed a significant effect of group during the 1^st^ (*F*2,31 = 3.90, *P* = 0.03) and 2^nd^ (*F*2,31 = 6.98, *P* = 0.003) trial performed during the retraining phase and the 2^nd^ trial (*F*2,31 = 4.11, *P* = 0.02) performed during the last day of training when all animals were tested with the transparent barrier. Additionally, the effect approached the level of significance during the first trial performed on the last day of training when all animals were tested with the transparent barrier (*F*2,31 = 3.17, *P* = 0.056). A post-hoc analysis (Fisher's test) revealed that the mice tested with the opaque barrier took a significantly more divergent path compared with the transparent group during the 1^st^ trial of the retraining phase (*P* = 0.03) and the semitransparent group during the 1^st^ (*P* = 0.02) and 2^nd^ trial (*P* = 0.001) of the retraining phase (Fig 7). In contrast, the mice tested with the opaque barrier took a significantly less divergent pathway compared with the transparent group during the 2^nd^ trial (*P* = 0.008) performed exclusively with the transparent barrier (Fig 7).

### Directional preference

**Fig 8:**
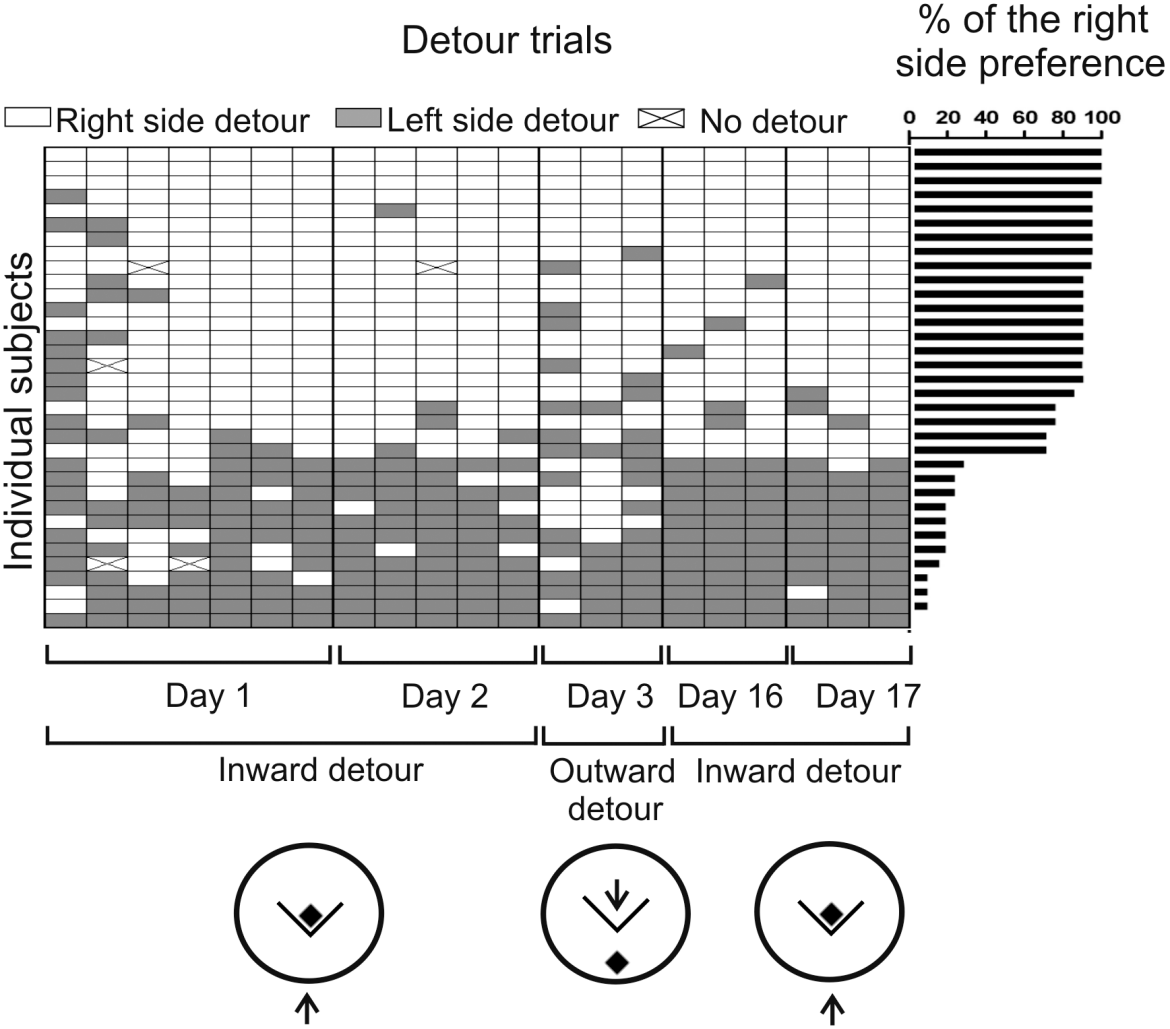
Preference for detouring around the right or left side of the barrier determined based on the body-centered coordinates. The arrow shows the starting point where the mouse was placed at the beginning of each trial. For more explanations see Fig 1.

All mice displayed a preference for detouring around the right (65%) or left (35%) side of the barrier preferentially using a strategy based on the body-centered coordinates (Fig 8). An individual preference ranged from 71 to 100% of detours made around the preferred side. Most mice (79%) maintained their preference for the left or right side determined based on the body-centered coordinates (Fig 1) during the outward detour trials when the position of the starting point and the target has been reversed (Fig 8). 47% of the mice maintained their preference (determined based on the body-centered coordinates) during all three outward trials, 32% maintained their preference during 2 out of 3 outward trials, while only 9% of the mice changed the direction during all outward trials.

## Discussion

We have found that an apparently simple task requiring the mice to move around a small barrier constitutes in fact a challenge that is strongly affected by the visibility of the target placed behind the obstacle (Fig 3) consistently with the previous results obtained in human infants [2], cotton-top tamarins [5] and some birds [6]. It is worth mentioning that all these experiments tested problem solving skills in naïve animals that were not familiarized with the barrier prior to the testing in contrast to studies examining path optimization in subjects that were familiarized with the barrier [33–35]. The increase in the latency to reach the platform (Fig 3) was associated with a sharp increase in the time spent in front of the transparent barrier (the measure of perseveration) in contrast to the semitransparent and opaque barrier (Fig 4). This shows that the sight of the target located behind the transparent barrier potently attracted mice and compromised their ability to find the solution. However, this was true only for mice that had no previous experience in detouring other barriers used in the experiment. Mice that learned to detour a semitransparent or opaque barrier also easily detoured the transparent barrier even during the first trial (Fig 2). These mice were also attracted by the sight of the target placed behind the transparent barrier as indicated by the change in the direction of movement during the initial approach to the barrier (Fig 7). However, the sight of the target did not impair detour performance of these mice as indicated by lack of differences in the latency between groups having different previous experiences (Fig 2). The obtained results show that the behavior of mice facing the problem of the transparent barrier depends both on the inhibitory skills that are necessary for curbing prepotent responses and on previous experiences constituting the basis for knowledge about spatial relationships between objects in the environment (physical cognition). The inhibitory skills played a major role at the time when mice had to find the proper solution but not in already experienced animals possessing basic detour skills. Therefore, we have recapitulated the key observations made previously in cotton-top tamarins [5]. It is also worth noting that there was large between-subject variability both in the level of perseveration and in the learning ability to solve the detour task (Fig 6). Previously, large individual variability in detour performance has been observed in chickens [4] and infant rhesus monkeys [36]. Köhler reported that “some particularly ungifted specimens keep on running up against the fence a long while even in the simplest predicaments” in contrast to other subjects [4]. This pattern of behavior resembled our observations in mice (Fig 6). Unfortunately, individual variability in cognitive skills is a frequently neglected issue although it constitutes an opportunity to discover mechanisms underlying biological processes [37]. In the past, scientists took an advantage of individual variability to uncover for example mechanisms of pain [38,39]. However, we are not aware of many similar reports emphasising individual differences in executive skills in mice. Nonetheless, the striking between-subject variability was recently observed in mice tested for cognitive flexibility and stability [40].

Our study also showed that the mice optimized the direction of movement based on previous experiences (Figs 5 and 8). The simplest strategy was to move toward the goal visible behind the barrier and next to swim along the wall separating the animal from the platform. The second possibility was to ignore the target and to swim directly toward the end of the barrier. Mice initially moved toward the target visible behind the barrier but changed the direction during training and chose a significantly more divergent pathway with respect to the goal (Fig 5). A similar pattern of behavior was also observed in the mice from the transparent group retrained after the rest period (Fig 7). This suggests that experienced mice planned in advance the direction of movement to optimize the path length. Such ability required the mice to remember the direction of movement and the outcome of the selected pathway and next to use this information during the subsequent trial. This means that the detour was not merely an execution of a learned sequence of movements. This conclusion is also supported by the behavior of mice during outward trials when the platform was placed 56 cm behind the barrier, in contrast to the inward trials performed with the platform touching the barrier. Most of the mice subjected to the first outward trial swam directly toward the platform after finding the edge of the barrier instead of turning and swimming along the wall toward the central part of the barrier where the platform was previously located. This again shows that the detour behavior of mice was not a simple sequence of learned movements. The finding that mice optimized the path length by the change in the direction of movement during the inward trials is important because the ability to take shortcuts was not previously reported in mice and there are scant reports showing such abilities in other species such as dingoes [1], dogs [41], rats [42], and hamsters [43,44]. Additionally, there are also some rat studies that employed a forced-choice paradigm to make an animal select a novel / shorter route [45,46]. However, it is worth noting that the behavior of an animal that has a free choice is affected by additional factors, such as a preference for familiar routes and minimization of the risk that are not found in case of the forced-choice paradigm [33].

The learning process led to gradual improvement as indicated by changes in latencies in all groups (Fig 3). It should be noted that the average latencies in mice tested with the transparent barrier (Fig 3) were strongly affected by the results obtained in two mice that learned to catch the edge of the barrier and stayed in this position for a prolonged period of time during the second day of training. However, this aberrant behavior disappeared during the training and finally all mice mastered the task. Recently, a similar situation was reported by Dettmer et al. [36] who observed an increasing individual variation across testing days in infant rhesus monkeys due to single behaviorally impaired subject. It also turned out that the improvement in the opaque group was slower than in the semitransparent one (Fig 3). Both the opaque barrier and the wall of the tank were painted white and the low contrast between objects could make it difficult for the mice to recognize borders of the barrier. A similar assumption has been made previously in case of experiments performed in wolves [10]. We painted the tank white because the EthoVision system is able to track animals only in a situation when there is a contrast between the subject and the background. In such a case a black barrier or barrier with conspicuous dark patterns may have made the task easier to learn. However, we were interested in behavioral changes induced by the visibility of the target and therefore we did not want to substitute the sight of the dark barrier for the sight of the dark platform. In such a case there would be no big difference between animals moving to the dark platform placed behind the transparent barrier and animals swimming toward the black barrier occluding the platform. Therefore, we used just a white barrier and this experimental design sufficed to show that the behavior of mice trained with the transparent and semitransparent barriers was indeed driven by the sight of the platform as indicated by differences between groups in time spent in front of the barrier and in the path direction.

The outward detour task enabled us to test a navigation strategy used by the mice to detour the barrier (Fig 1). There are two main strategies that can be applied to navigate in the environment: the allothetic (allocentric) strategy based on the position of environmental landmarks (place learning) and the egocentric strategy based on the body-centered coordinates (response learning) [47,48]. These two strategies can be distinguished when a position of the starting point and the goal is reversed [48,49] (Fig 1). First, we found that all mice displayed a preference for turning right or left during inward trials. Second, most of the mice (79%) maintained their preference for the left or right side defined by the body-centered coordinates during outward trials (Fig 8). It means that most of the mice followed their respective left or right side (egocentric strategy) instead of using landmarks to navigate during the task. In contrast, more dingoes tested on the detour task [1] changed the preference for their respective left or right side and travelled along the same side of the barrier also in case of a reversed condition. This suggests that dingoes used landmarks more frequently than a body-based frame of reference to navigate during the detour task [1]. A preference for one side of the barrier has also been found in dogs and quokkas [9,11] but the mode of navigation in these species was not reported and therefore there are no other detour studies for comparison. It should be noted that the navigation strategy is affected also by the amount and characteristic of the extra-maze cues, and animals may use either an allothetic or egocentric strategy depending on the surrounding of experimental arena [48]. Therefore, the preference for one side of the barrier, which was observed in various species, may be a more consistent behavioral trait than preference for an allothetic or egocentric strategy.

An important question is how the mice acquired the detour skills. During the first trial, there was a sharp increase in the latency in all groups and the mice needed at least several trials to master the task (Fig 3). At the beginning, the behavior was characterized by a repeated pattern of swimming toward the barrier and away until the mice found the open side of the platform (Fig 6a and 6b; S1 File and S1 Fig). Furthermore, the mice detoured the barrier from both sides during initial trials and the preference for one side developed during the course of training (Fig 8). These findings suggest that naïve mice did not plan the strategy to detour the obstacle during initial trials and that they reached the platform based on the trial-and-error method (repeated pattern of swimming toward the barrier and away). However, the initial strategy based on random search changed during the course of training as indicated by a strong preference for one side of the barrier (Fig 8) and the path optimization (Fig 5). It should be noted that obtained data do not allow dissecting precisely the contribution of trial-and-error learning from executive skills. A precise understanding of the mechanism underlying the detour behavior of mice will require further research. Translation of human cognitive tasks to animals is associated with a possibility that there are various explanations of animal behavior including accounts that are simpler than originally expected [50]. However, irrespective of obtained data, such comparative studies stimulate research advancing our understanding of behavior [50,51].

Our study also shows also that there is a need to pay attention to proper classification of barriers used in detour experiments. The term “transparent barrier” is commonly used in the literature also in case of barriers that are made from bars, grids or latticed screens [1,34,35,52]. These barriers are comparable to our semitransparent barrier that constituted the easiest task in contrast to the truly transparent barrier that constituted the most difficult task. Two main factors may be responsible for differences in performance between animals tested with the semitransparent and transparent barrier. First, the effect of target visibility is stronger in case of truly transparent barriers. Second, animals faced with the problem of truly transparent barriers have to resolve a conflict between senses (vision vs. tactile perception), while this challenge is not present in case of semitransparent barriers. This problem is not well recognized because there are no other studies comparing completely transparent and semitransparent barriers in mammals. Nonetheless, our results are consistent with the previous study performed in birds that also revealed large differences between transparent and semitransparent barriers [6]. Furthermore, Zucca et al. [6] found differences between groups of birds trained with semitransparent barriers covered with various patterns such as vertical and horizontal bars. Therefore, studies employing barriers made of different materials should be compared cautiously.

Furthermore, there are also other problems that make direct comparisons between species difficult. For example, differences in learning may result from various levels of motivation associated with different experimental designs (food reward, water reward, water or pain avoidance) applied to species differing in size, metabolism, sensory capacity, and ecology. Therefore, quantitative differences between various species may not be reliable in case of behavioral studies.

The development of a mouse model of the detour test should enable the usage of rodents to study biochemical and anatomical mechanisms of cognitive functions that were previously investigated only in primates [12,53,54]. Furthermore, monkeys are used in preclinical studies employing the detour test to study cognitive effects of various drugs [55,56]. Previously, pharmacologically-induced dysfunction in detour skills have been used to model cognitive impairments observed in human psychiatric [19,20,23,57] and neurological diseases [15–17]. The detour test was also used to study the effect of viral infection [58] and early life stress on cognition in monkeys [59]. Substitution of rodents for primates would be beneficial from the ethical point of view and this is especially important because of the currently advocated 3Rs principle (replacement, reduction and refinement of animal use) [60]. Furthermore, experiments performed in mice enable researchers to study the effect of genetic manipulations on cognitive performance and the interaction between genes and drugs. Therefore, mice offer research opportunities that are not currently possible to achieve when primates are used.

## Conclusions

We have found that an apparently simple task requiring mice to move around a small barrier constituted in fact a challenge that was strongly affected by the visibility of the target.

The detour behavior depended both on the inhibitory skills that are necessary for curbing prepotent responses and on previous experiences constituting the basis for knowledge about spatial relationships between objects in the environment. An improvement in performance depended on the learning process as evidenced by decreased latencies and changes in the direction of movement during subsequent trials. Finally, all mice displayed a preference for one side of the barrier and most of them relied on the egocentric strategy. The obtained results show for the first time that behavior of mice subjected to the detour task is comparable to the behavior of other mammals tested previously. Reported findings are important because a detailed characterization of the detour behavior in mice constitutes the first step toward the substitution of rodents for primates in laboratory experiments investigating biological and pharmacological mechanisms underlying detour skills.

## Supporting Information

S1 Fig An example of tracks from consecutive trials of the detour test. The track denotes an animal’s path recorded by EthoVision system based on the position of the mathematical centre of tracked object. Tracks are shown against a single background image.
(PDF)

S1 File Video presenting the detour test. (MPG)

S2 File Datasets. *—mice removed from the final analysis because of an error in changing the barrier that occurred at the beginning of the training.
(XLSX)
