# Supplementary material for: Detour Behavior of Mice Trained with Transparent, Semitransparent and Opaque Barriers
Source: PLoS One. 2016 Sep 2;11(9):e0162018. doi: 10.1371/journal.pone.0162018 (PMC5010287; doi:10.1371/journal.pone.0162018)
Supplement: S1 Fig — The track denotes an animal’s path recorded by EthoVision system based on the position of the mathematical centre of tracked object. Tracks are shown against a single background image. (PDF) [file pone.0162018.s001.pdf]

S1 Fig. Animals belonged to one litter and were trained in the order from mouse 1 to 5.

Session	**Habituation**				
	Transparent barrier		Semitransparent barrier		Opaque barrier
	Mouse 2	Mouse 5	Mouse 1	Mouse 4	Mouse 3
1	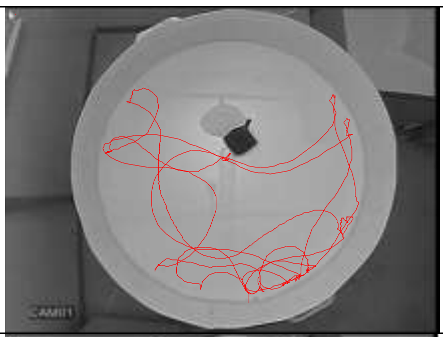	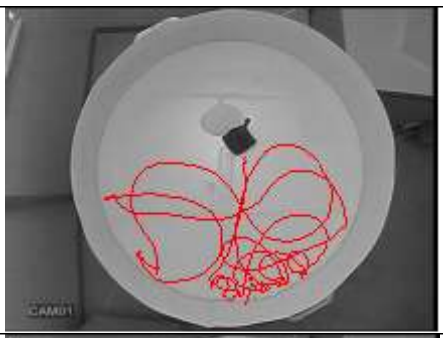	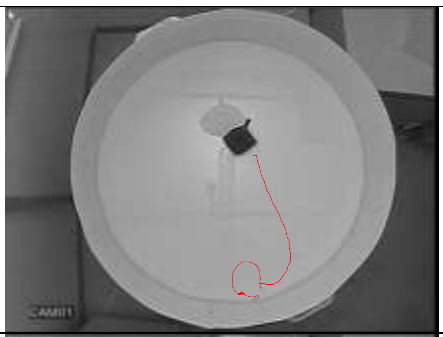	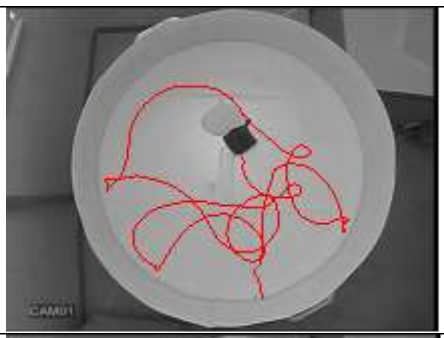	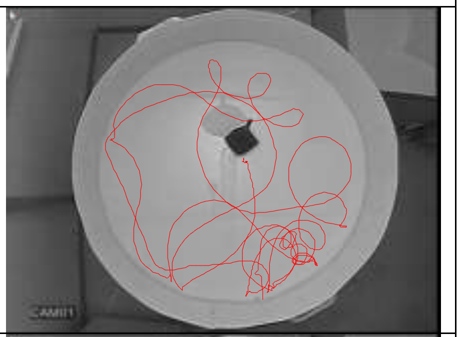
2	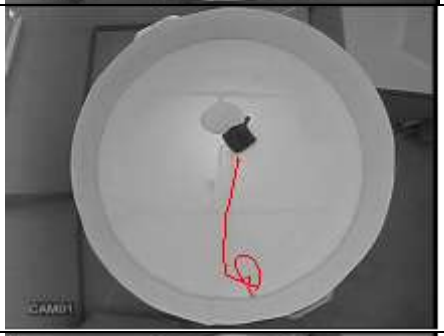	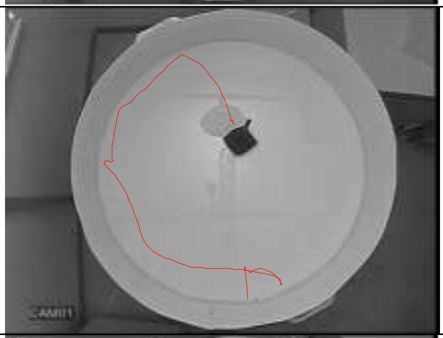	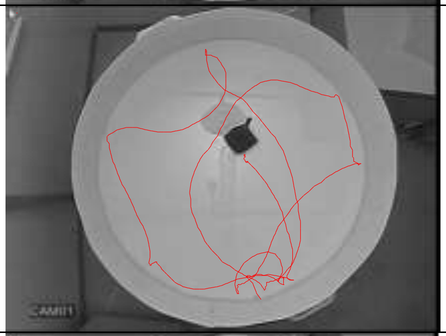	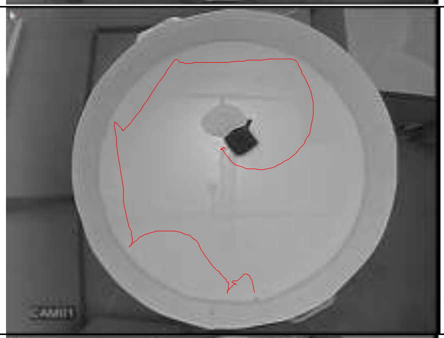	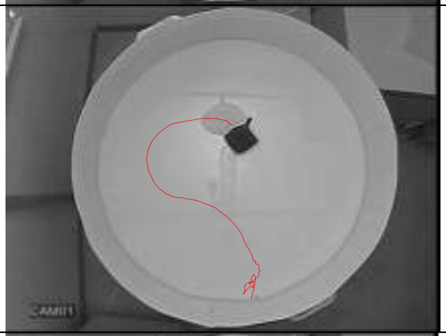
3	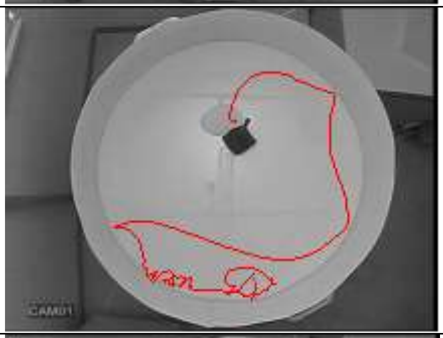	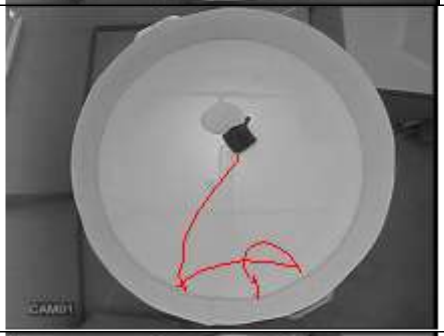	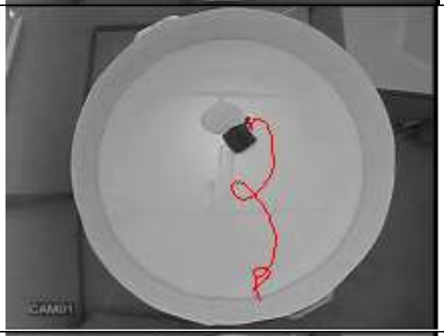	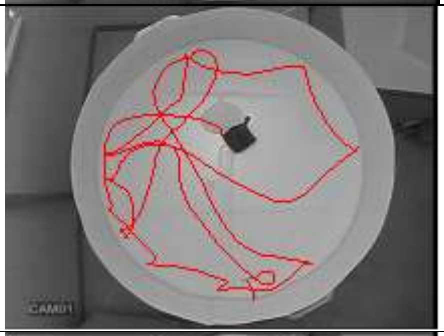	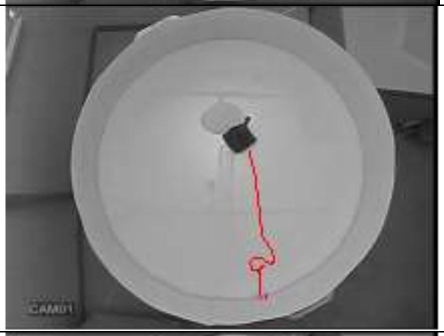
4	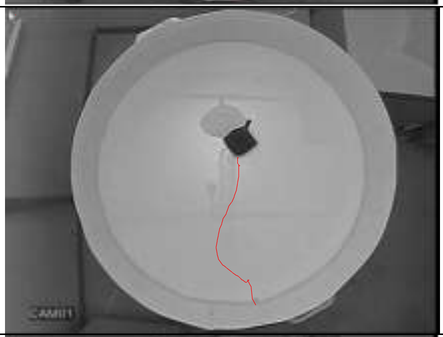	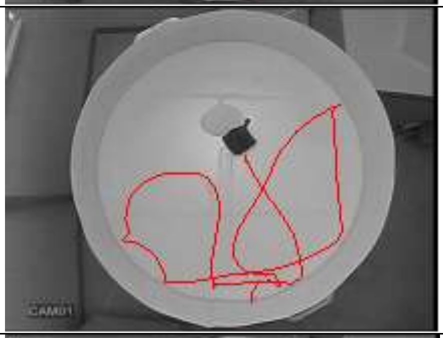	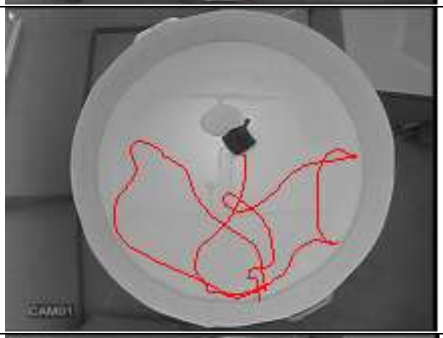	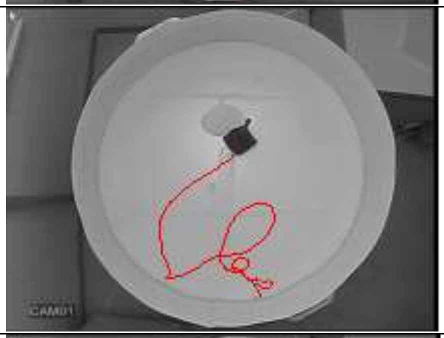	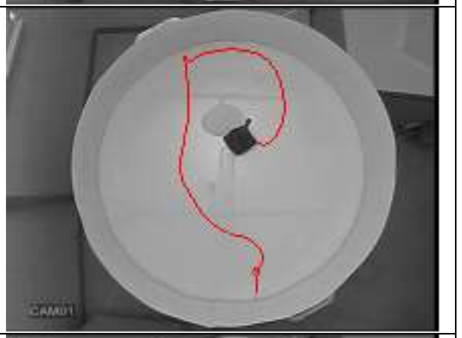
5	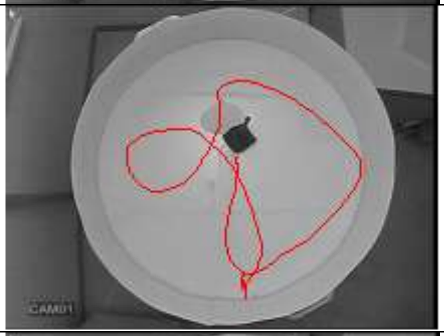	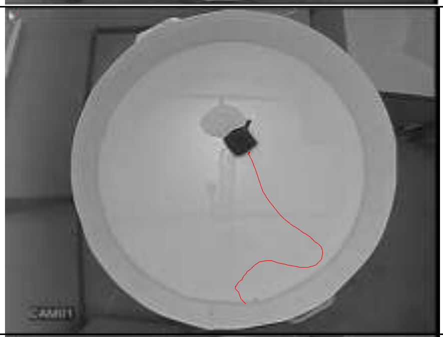	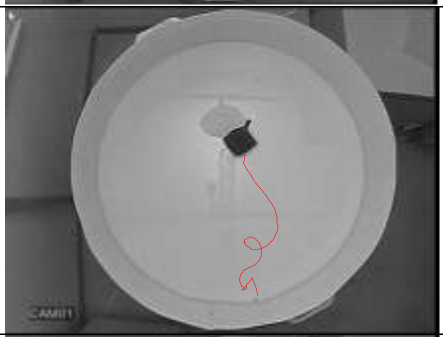	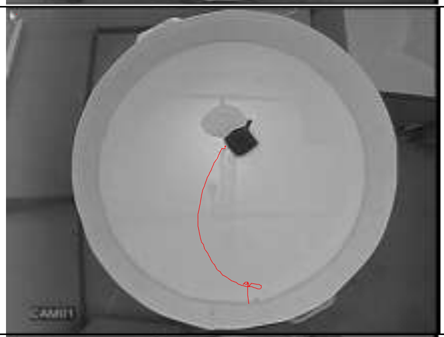	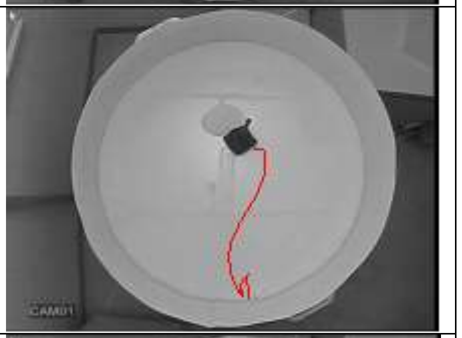
6	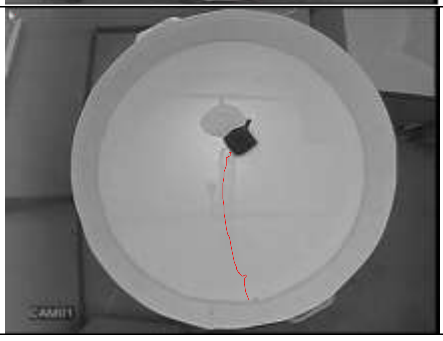	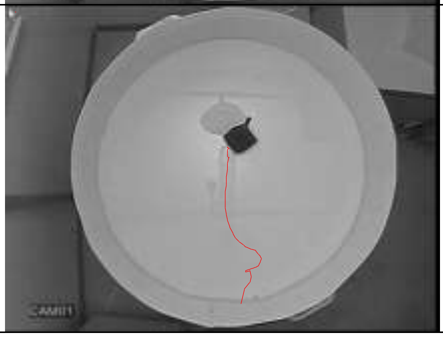	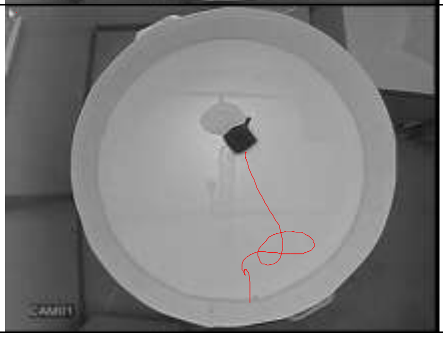	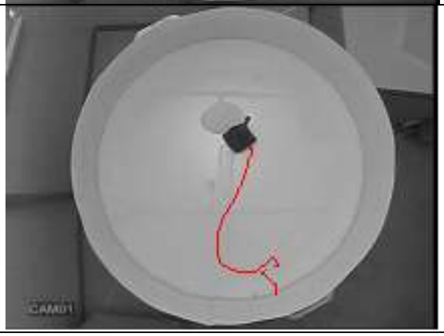	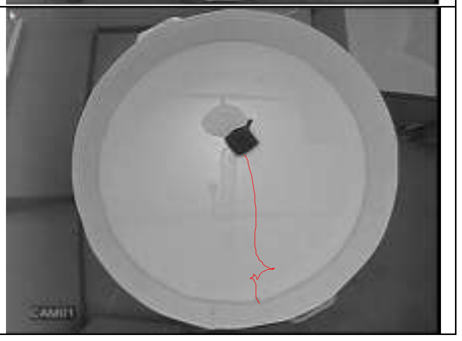

Trial	Inward detour (Day 1)				
	Transparent barrier		Semitransparent barrier		Opaque barrier
	Mouse 2	Mouse 5	Mouse 1	Mouse 4	Mouse 3
1	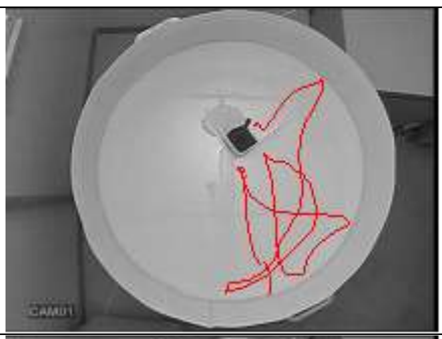	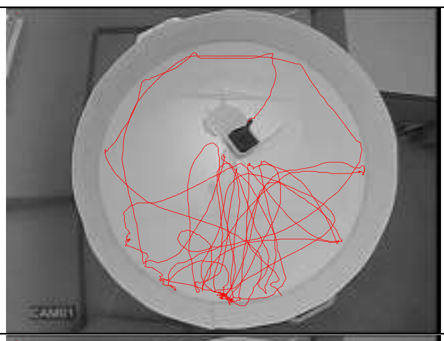	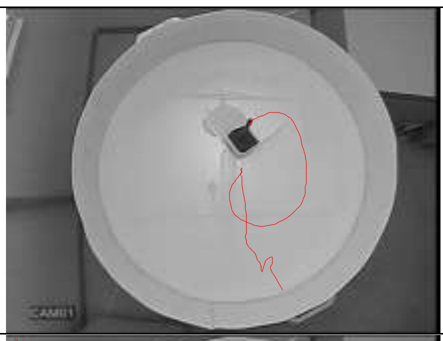	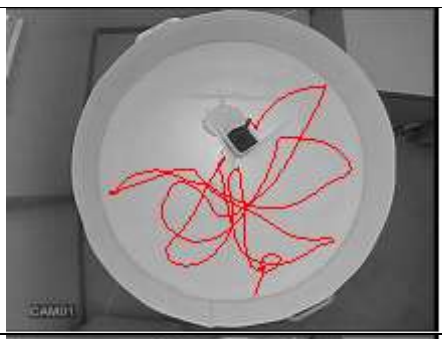	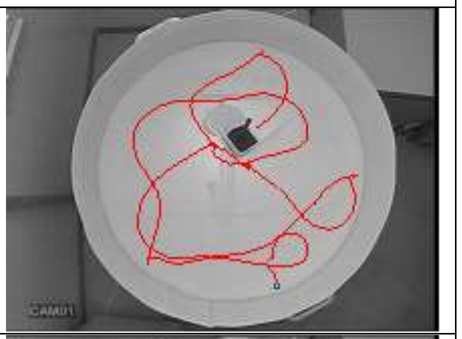
2	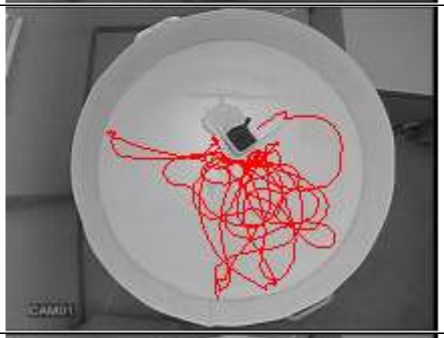	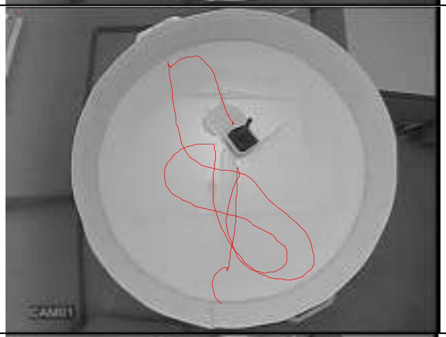	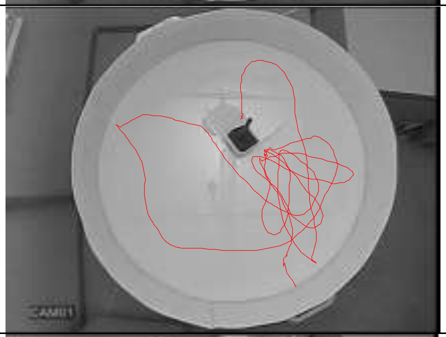	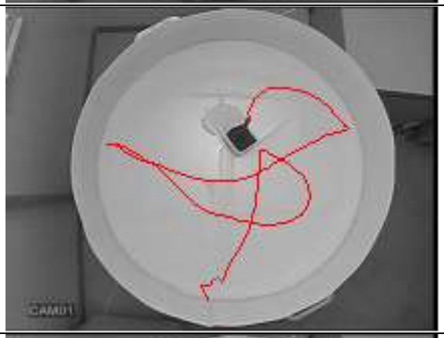	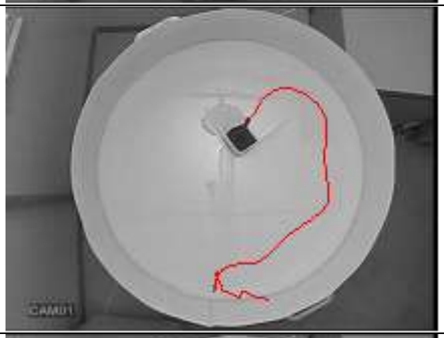
3	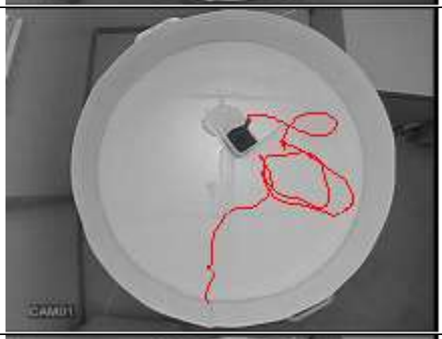	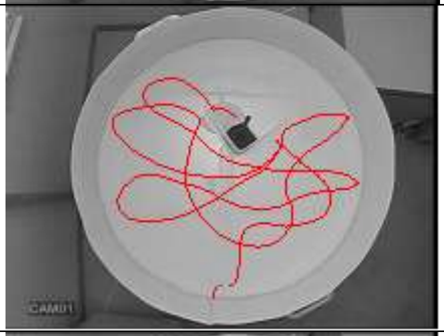	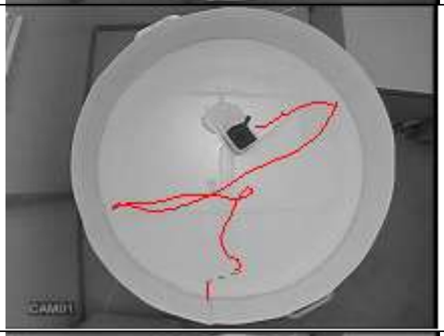	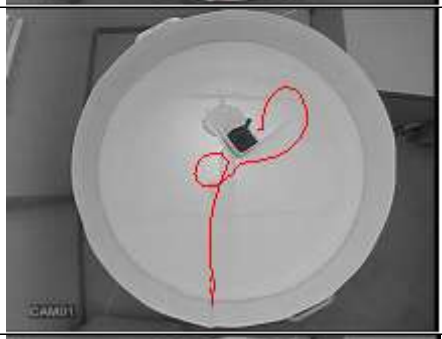	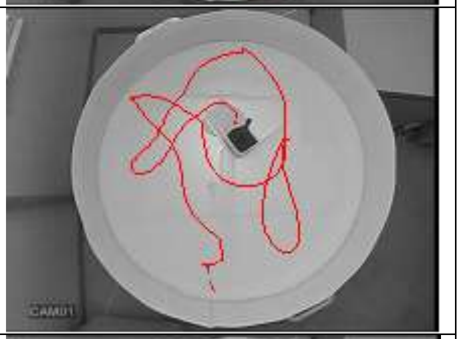
4	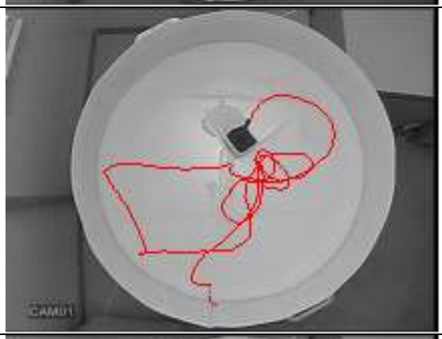	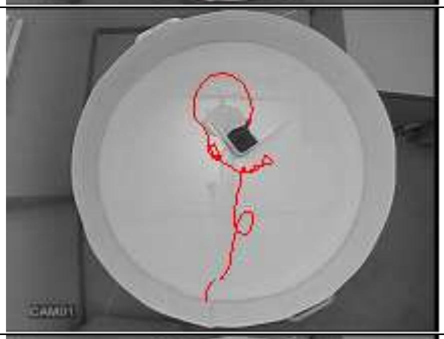	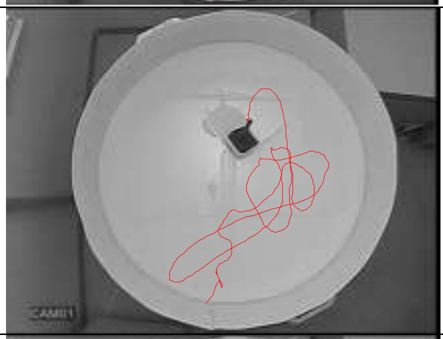	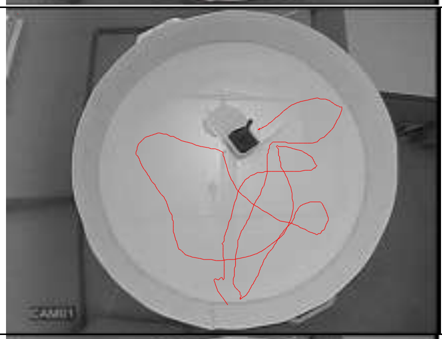	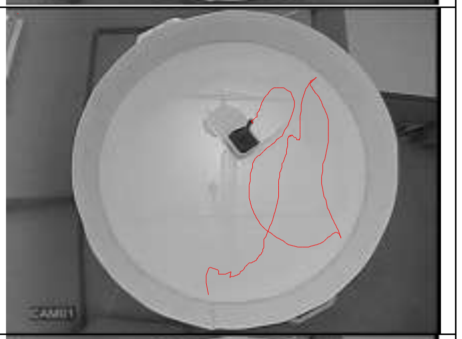
5	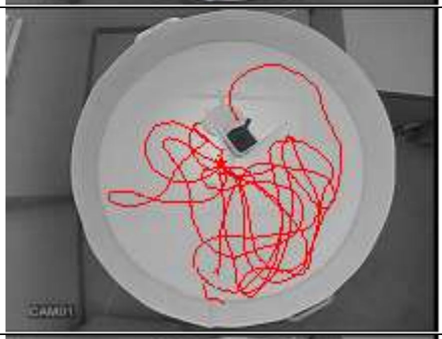	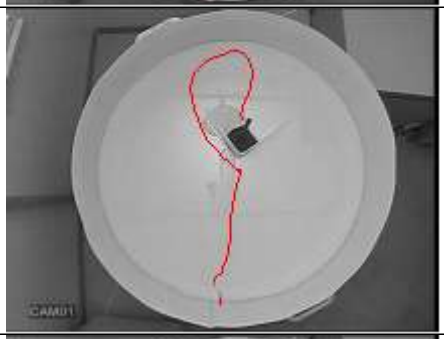	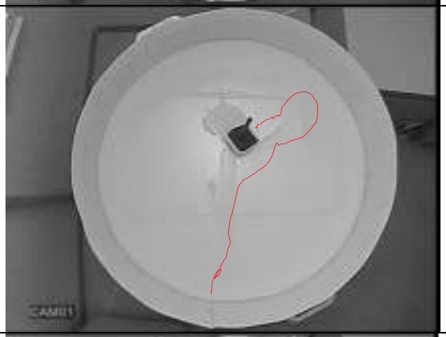	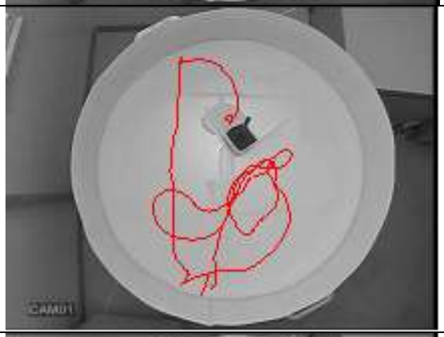	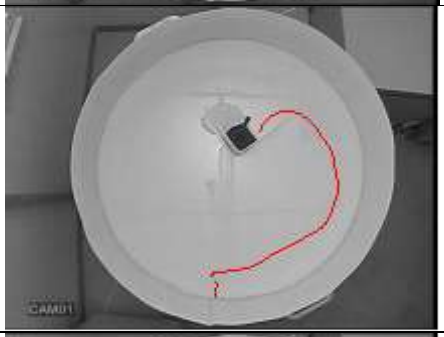
6	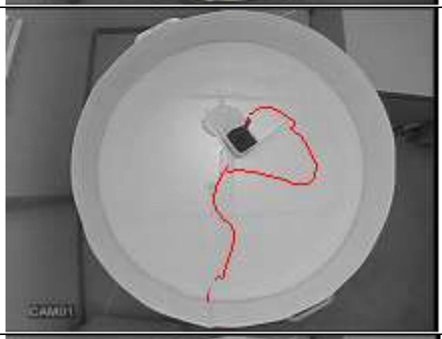	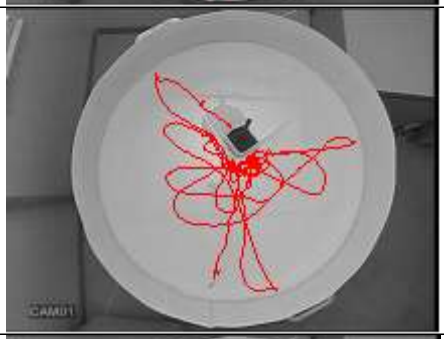	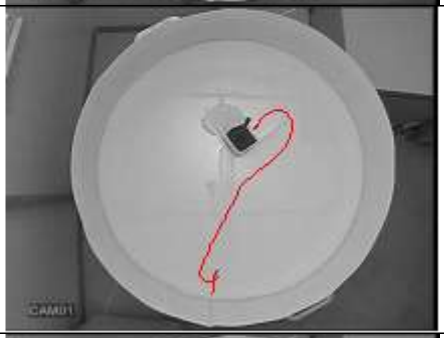	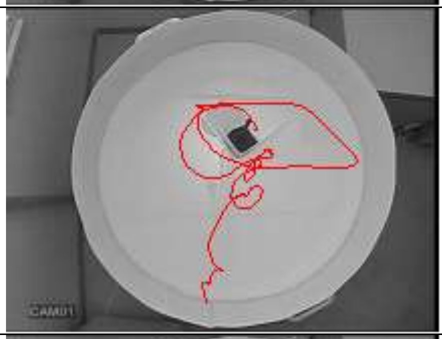	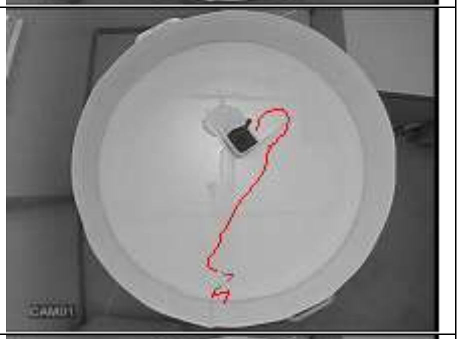
7	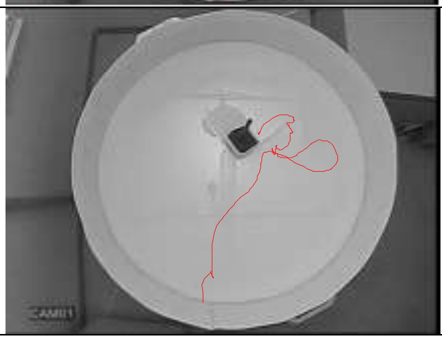	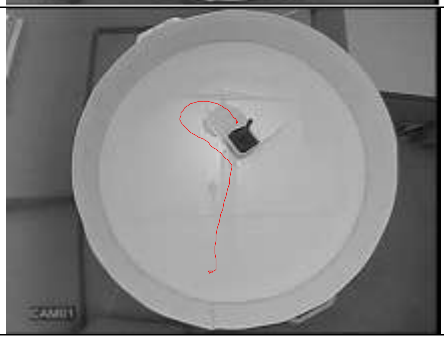	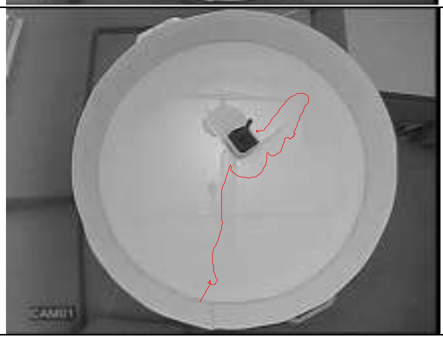	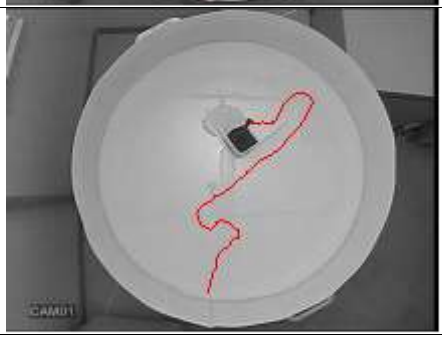	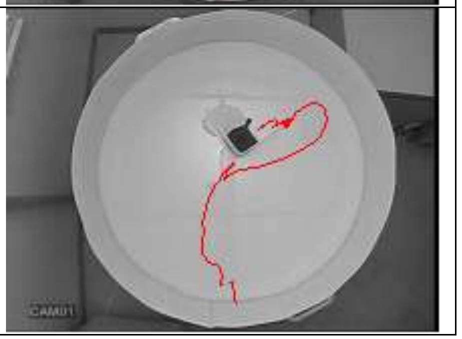

Trial	**Inward detour (Day 2)**				
	Transparent barrier		Semitransparent barrier		Opaque barrier
	Mouse 2	Mouse 5	Mouse 1	Mouse 4	Mouse 3
1	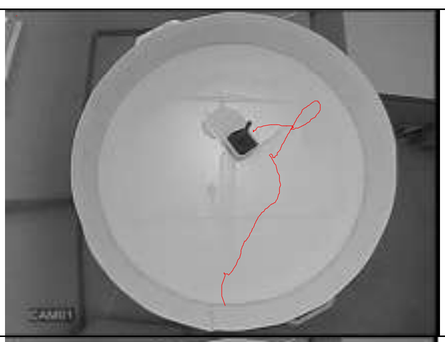	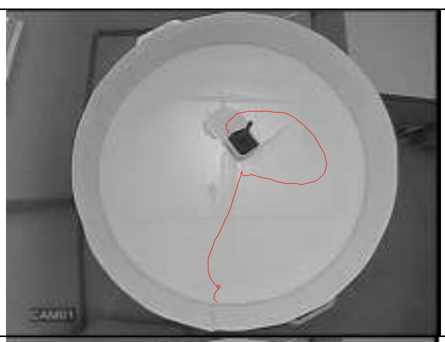	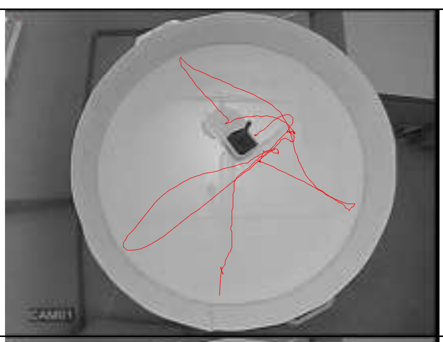	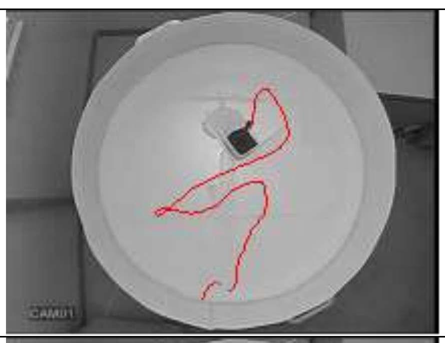	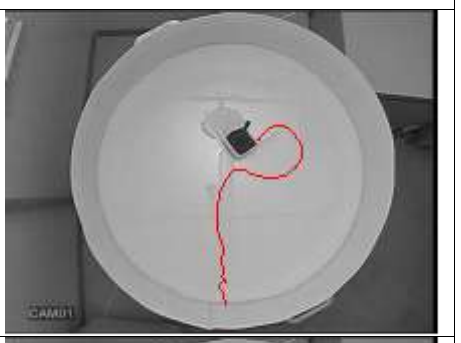
2	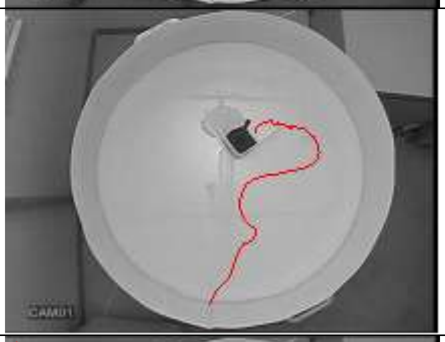	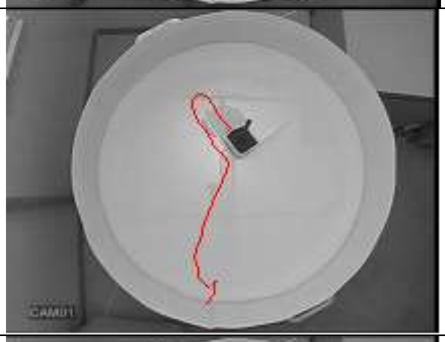	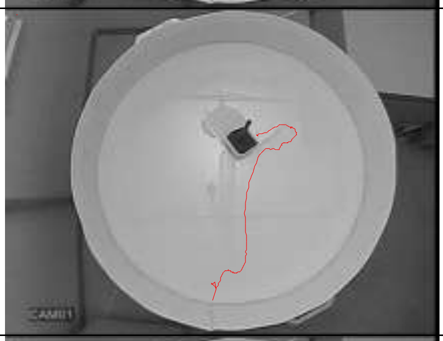	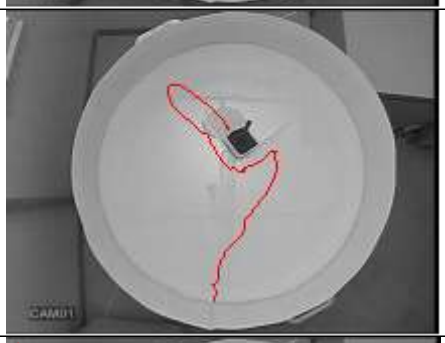	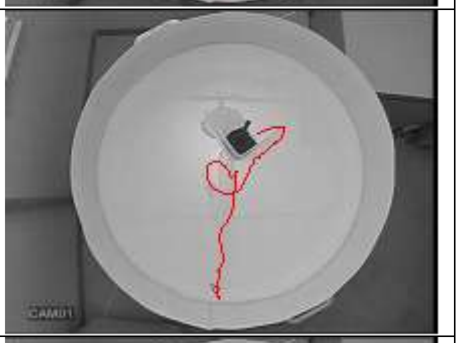
3	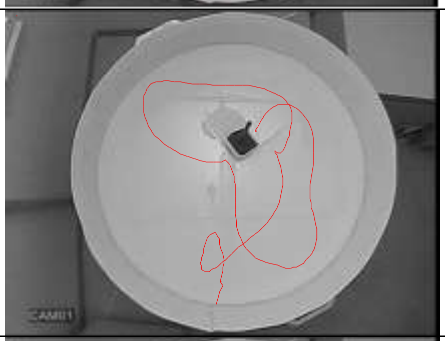	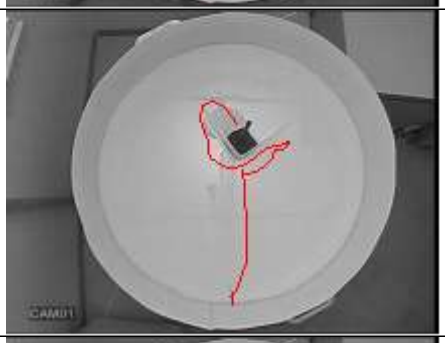	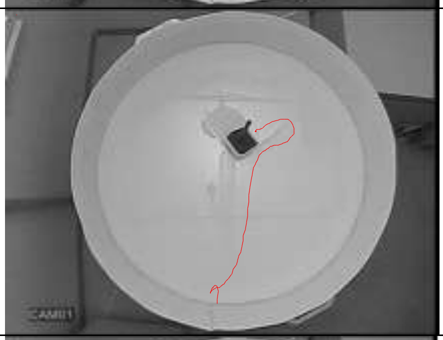	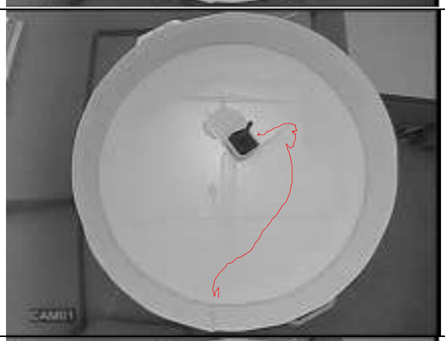	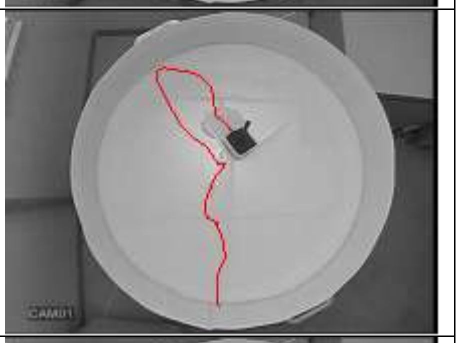
4	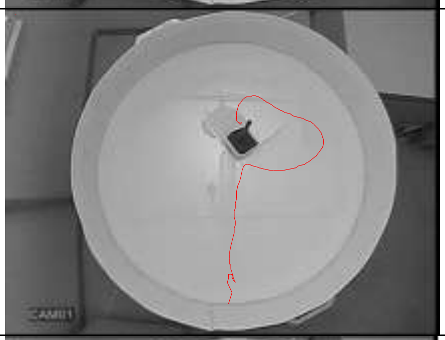	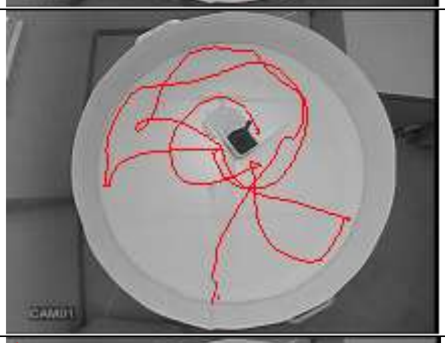	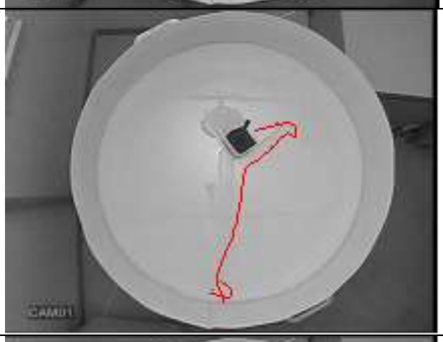	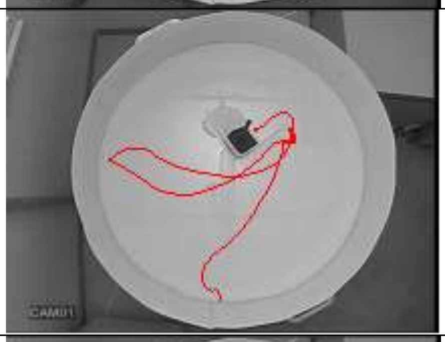	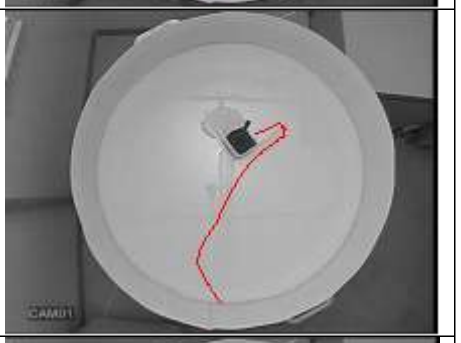
5	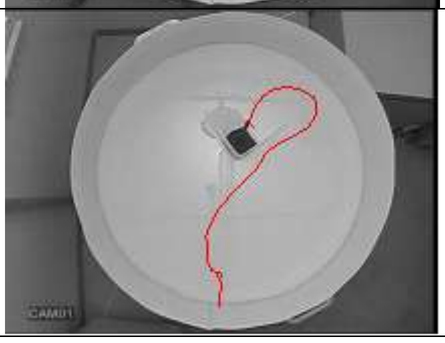	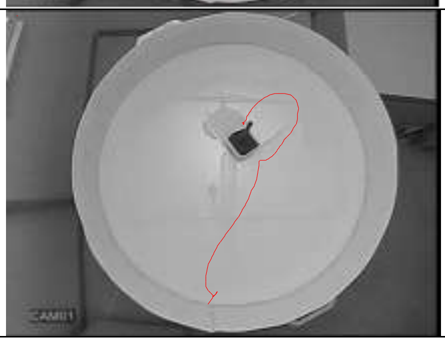	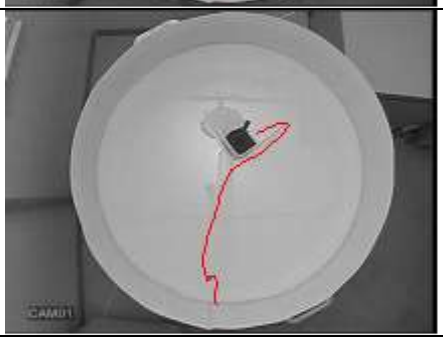	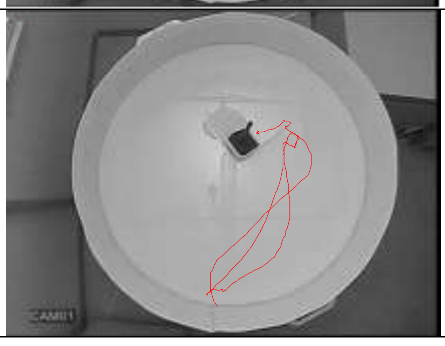	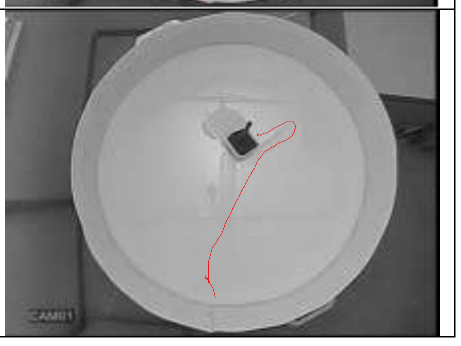

Trial	**Outward detour (Day 3)**				
	Transparent barrier		Semitransparent barrier		Opaque barrier
	Mouse 2	Mouse 5	Mouse 1	Mouse 4	Mouse 3
1	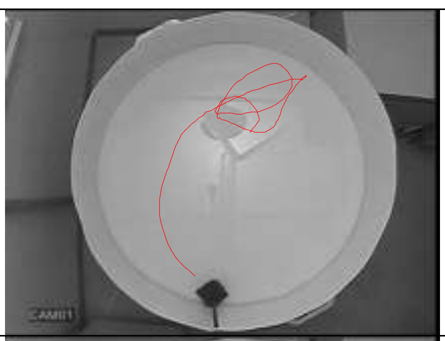	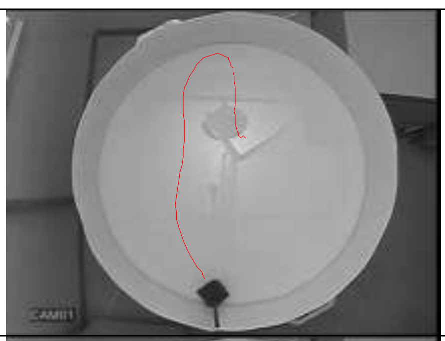	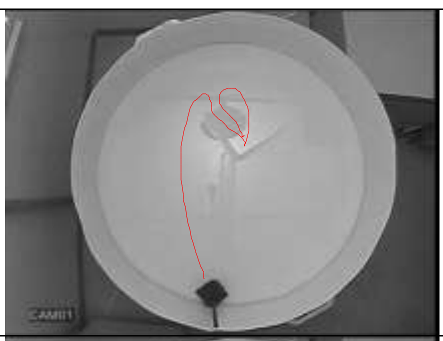	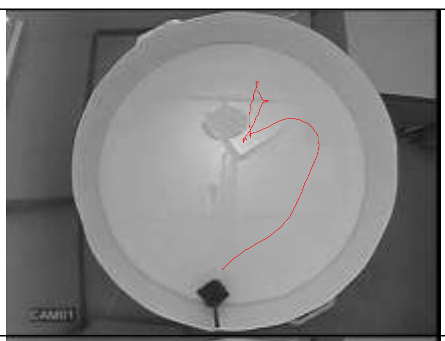	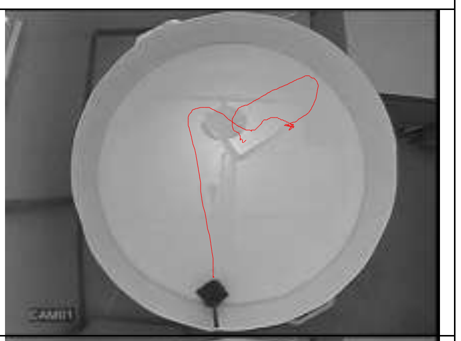
2	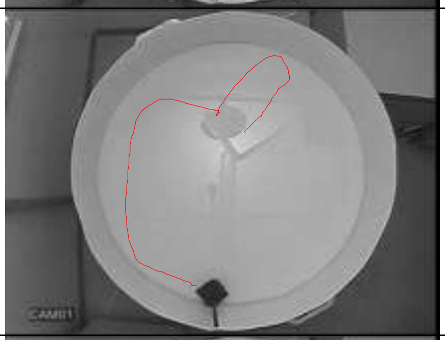	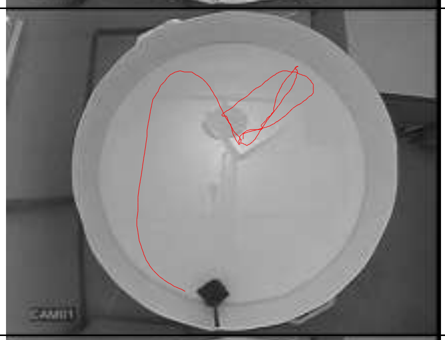	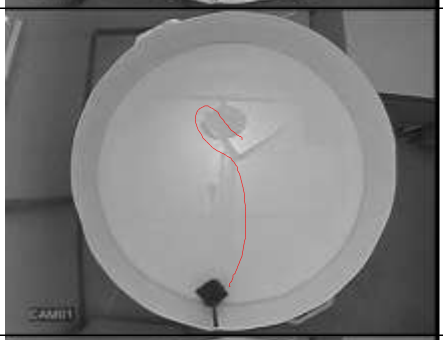	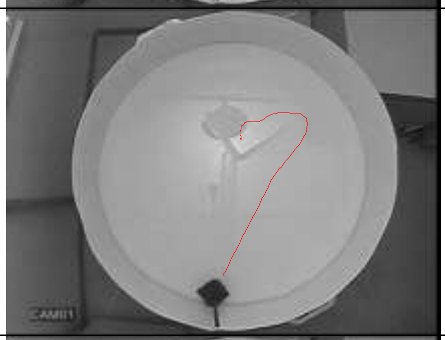	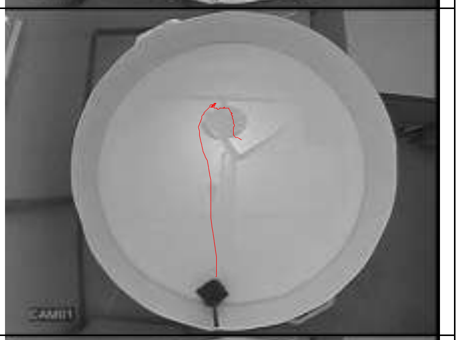
3	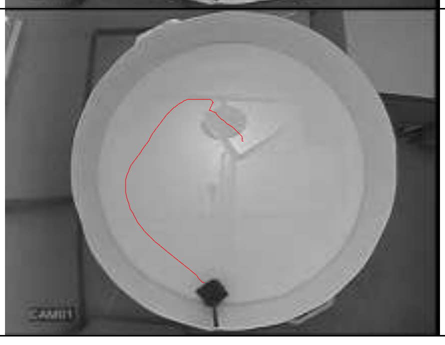	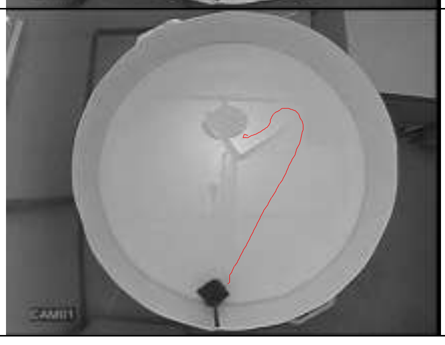	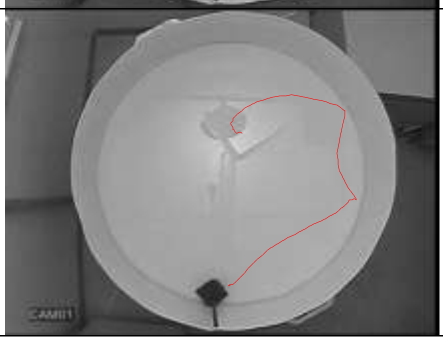	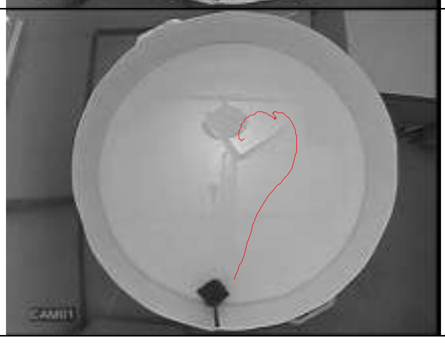	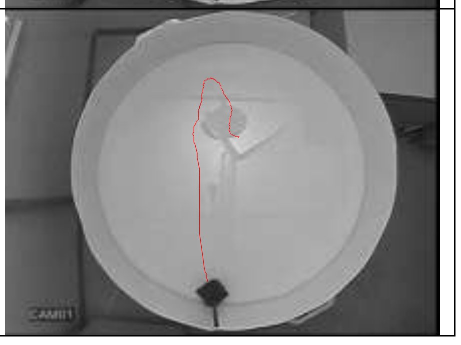
